# Experimental Study on the Kinetics of CO_2_ and H_2_O Adsorption on Honeycomb Carbon Monoliths under Cement Flue Gas Conditions

**DOI:** 10.1021/acssuschemeng.1c07213

**Published:** 2022-01-31

**Authors:** Nausika Querejeta, Fernando Rubiera, Covadonga Pevida

**Affiliations:** Instituto de Ciencia y Tecnología del Carbono, INCAR-CSIC, c/ Francisco Pintado Fe 26, 33011 Oviedo, Spain

**Keywords:** CO_2_ and H_2_O adsorption, kinetics, carbon monoliths, cement flue gas

## Abstract

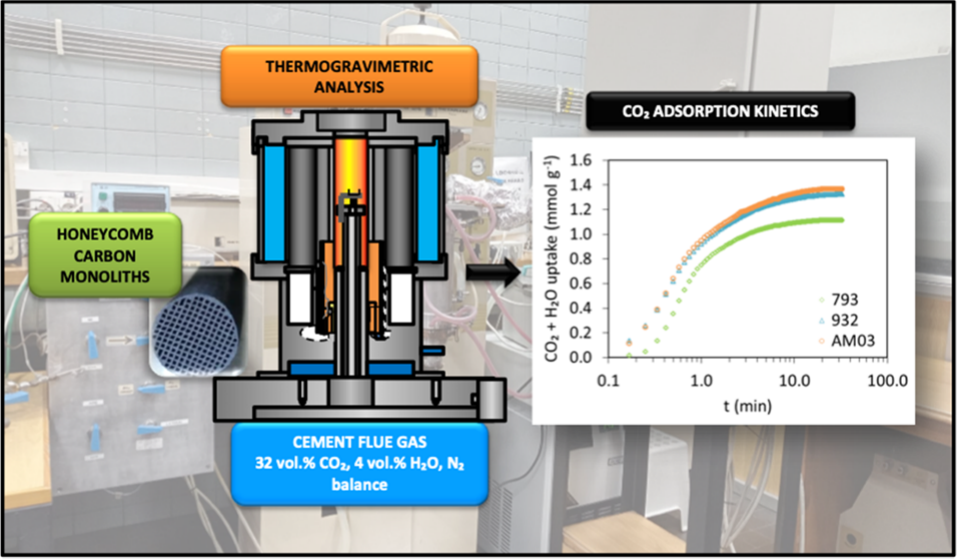

The main challenge of adsorption consists in the production of materials that can be used in real situations. This study comprehensively describes the CO_2_ and H_2_O adsorption behavior of honeycomb-shaped sorbents commonly used in rapid pressure swing adsorption cycles (RPSA). With this purpose, the kinetics and equilibrium of adsorption of CO_2_/H_2_O/N_2_ mixtures on three honeycomb carbon monoliths (793, 932, and AM03) were assessed in a thermogravimetric analyzer (TGA) under different postcombustion capture scenarios (temperature of 50 °C and several concentrations of CO_2_). The kinetics study exhibited that the single adsorption of CO_2_ and H_2_O can be adequately described by the Avrami and exponential decay-2 models, respectively. As expected, the three carbon monoliths presented fast adsorption of CO_2_ from a CO_2_/H_2_O mixture. Furthermore, when humid flue gas was considered, overall adsorption kinetics were governed by CO_2_. Besides, the experimental data fitting to the intraparticle diffusion model showed that gradual CO_2_ and H_2_O diffusion toward the micropores was the rate-limiting stage. The obtained results give a better insight into the selective adsorption of CO_2_ and the potential of honeycomb carbon monoliths to separate CO_2_ from humid flue gas in the context of the cement industry. Carbon monolith 793 is the best carbon monolith candidate to capture CO_2_ under the evaluated conditions: a capacity of adsorption of 1 mmol of CO_2_ g^–1^ and favorable kinetics in 32 vol % CO_2_ and 4 vol % H_2_O_(v)_, at 50 °C and 101.3 kPa.

## Abstract 2

Adsorption equilibrium and kinetics of CO_2_/H_2_O/N_2_ mixtures on three honeycomb carbon monoliths were assessed in a thermogravimetric analyzer under different scenarios representative of postcombustion capture.

## Introduction

Among the industrial sources, cement plants constitute a major CO_2_ emitter.^1^ The CO_2_ emissions from the cement industry are in the order of 1306 Mt year^–1^, around 27% of the carbon emissions from industry. It implies that for every tonne of cement produced, 0.6–1.0 t of CO_2_ are emitted.^2^ Half of the emitted CO_2_ results from the calcination of limestone; the combustion of different fuels in the kiln such as coal, petroleum coke, tires, waste oil, sewage sludge, etc., account for an additional 40%; and transportation and electricity used in manufacturing operations contribute 5% each.^3^

CO_2_ capture, utilization, and sequestration (CCUS) technologies will be crucial to reduce CO_2_ emissions from the cement sector, specifically process emissions associated with limestone calcination.^4^ Postcombustion CO_2_ capture technologies similar to those applied in the power sector are the starting options and can easily be retrofitted to the existing facilities, therefore reducing the time frame for large-scale deployment.^5^

Solid-based technologies to capture CO_2_ entail an alternative to the benchmark chemical absorption relying on their capability to be more energy-efficient. Gas purification and impurity removal can be carried out by adsorption, which is a well-implemented process in the chemical and petrochemical industries. In any low-temperature adsorption-based process, the sorbent selectively separates the adsorbate molecules from a gas mixture in which the pair adsorbate–sorbent establishes the nature of the type of bonding involved.^6,7^ Under postcombustion CO_2_ capture conditions, the presence of water vapor is a nuisance to the selective capture of CO_2_ by a physical adsorbent. The competition of H_2_O against CO_2_ for the adsorption sites in the solid leads to a loss of adsorption capacity. The extent of the impact of water vapor on CO_2_ uptake depends on both the relative humidity (RH %), the concentration levels of CO_2_ in the gas streams,^6^ and the relative kinetics of CO_2_ and H_2_O adsorption.^8^

Accurate knowledge of multicomponent adsorption equilibria and kinetics is essential to comprehend the effects of moisture content on the CO_2_ adsorption from flue gases. It requires a comprehensive understanding of single and multicomponent adsorption thermodynamics to describe the equilibria of gas components and water vapor. In addition, kinetics provides knowledge about the adsorption rate under established conditions. Thus, both kinetic and equilibrium data constitute very useful information to ensure the successful removal of CO_2_ from industrial gases through the utilization of a suitable adsorbent.^9,10^

Several studies have been published in the literature dealing with CO_2_ and H_2_O single-component adsorption kinetics. For instance, Wei et al.^11,12^ studied the kinetics of the adsorption of CO_2_ using activated carbon produced from waste ion-exchange resin. After employing a broad interval of temperatures and pressures, they concluded that the models that most appropriately described the CO_2_ adsorption kinetics were those of Avrami and fractional order. Likewise, Zhang et al.^13^ investigated the H_2_O_(v)_ kinetics of adsorption at different temperatures and under different relative humidity conditions on the inner surface of silica-based nanoporous materials. The exponential-decay-2 model was found to satisfactorily fit the kinetic data for water vapor adsorption. In terms of binary CO_2_/H_2_O adsorption, authors such as Li et al.^14,15^ performed breakthrough experiments using activated alumina F-200 and CDX (a special BASF mixture made of alumina and NaY) to explore the impact of CO_2_ and H_2_O on each other over a wide range of concentrations of saturated water vapor and at different temperatures. These authors found that at increasing humidity, the inhibitory effect of CO_2_ on H_2_O diminished and the H_2_O adsorption quickly recovered the single-component adsorption values. For alumina CDX, the CO_2_ loading under dry conditions strongly decreased from 2.3 to 0.3 mmol g^–1^ at a relative humidity of 2.79% (0.12 kPa of water vapor). Conversely, the amount of CO_2_ adsorbed in the H_2_O_(v)_/CO_2_ system remained the same; however, a mild decrease was observed for higher humidity levels. Likewise, Xu et al.,^16^ using activated carbon GC1200, observed, at the same partial pressure, that both CO_2_ and H_2_O slightly diminished their loadings when mixed with regard to their pure component loadings.

The above-mentioned research has provided a better insight into the mechanism of adsorption and an effective way to predict the CO_2_–H_2_O adsorption behavior. More recently, in previous work,^6^ we evaluated the effect of relative humidity (RH %) on the CO_2_ uptake of a potassium-based sorbent under simulated flue gas conditions and concluded that a relative humidity of around 20% in the K_2_CO_3_-doped biocarbon bed benefited the carbonation reaction and increased the CO_2_ uptake, which showed a value of 1.9 mmol g^–1^ at 50 °C and 14 kPa CO_2_. This was achieved regardless of the H_2_O flue gas concentration. Furthermore, at higher temperatures between 300 and 500 °C, Coenen et al.^17^ developed a kinetic model, based on an extensive thermogravimetric analysis (TGA) study, that was able to represent the CO_2_ and H_2_O adsorption and desorption kinetics on a potassium-promoted hydrotalcite-based sorbent. The model took also into account their complex interactions. However, as far as we know, a comprehensive kinetic study of the adsorption behavior of CO_2_, H_2_O, and their interactions on activated carbons has not been reported as yet.

Flue gas from the cement industry usually contains a higher CO_2_ concentration of ca. 14–33%, compared to the 12–14% and 4% CO_2_ for pulverized fuel (pf) coal and gas power plants, respectively.^3,18^

The main challenge of adsorption, when applied at an industrial scale, consists in the production of materials that can successfully handle real process conditions. The adsorption capacity should not be the sole criteria for decision-making and process costing. The efficiency of the process and the technological feasibility should be balanced as indicated by Sun et al.^19^ For instance, adsorption/desorption kinetics impact the effectiveness of the process and the associated expenses. Better design and integration of the adsorption and desorption units can increase the speed of the cycles, but this can induce some problems in process engineering and, eventually, give rise to limitations in the selection of adsorbents. As indicated by Querejeta et al., carbon-based adsorbents are selective to CO_2_, can be regenerated without difficulty, and contrarily to other adsorbents (i.e., zeolites or metal–organic frameworks (MOFs)), they are hydrophobic and present high stability under humid environments.^20^ Besides, the use of monolith-structured carbon-based solid sorbents with flow-through channels, such as the honeycomb-shaped sorbents selected in this study, is very suited for rapid pressure swing adsorption cycles (RPSA). The monolith-structured sorbents allow high flow rates of gases carrying dust without experiencing the associated pressure drop of packed beds, avoid the fluidization of adsorbents in fluidized beds, offer a larger geometric surface area that enhances the contact between gas and solid, and are easily scalable. Nevertheless, the open structure of honeycomb monoliths may result in poor adsorption performance.^21,22^

With this regard, the equilibrium and kinetics of adsorption of CO_2_/H_2_O/N_2_ mixtures on three honeycomb carbon monoliths were studied in a thermogravimetric analyzer (TGA). The findings of this research give a better comprehension of the selective adsorption of CO_2_ and the potential of honeycomb carbon monoliths to separate CO_2_ from humid flue gas in the context of the cement industry.

## Characterization of Adsorbents

In this study, three honeycomb carbon monoliths, denoted as 793, 932, and AM03, were evaluated in CO_2_ adsorption experiments. These monoliths were produced by MAST Carbon International Ltd. avoiding the addition of a binder. The process entailed the carbonization of extruded phenolic resins and their ulterior activation. This method of fabrication gives rise to a singular and precise control of the monoliths structure at the micro and macropore levels.^22^

### Textural Properties

Characterization of the porosity of the activated carbon monoliths was accomplished by N_2_ adsorption isotherms at −196 °C (Micromeritics ASAP 2010), and CO_2_ adsorption isotherms at 0 °C (Micromeritics TriStar 3000). Before the measurements, the samples were degassed at 100 °C overnight under vacuum.

The porous texture of the samples is well described by N_2_ and CO_2_ adsorbates. N_2_ adsorption isotherms include relative pressures that span from *p*/*p*^0^ = 0 to 0.98 and cover a wide range of pore sizes, but N_2_ suffers diffusion limitations in ultramicropores (<0.7 nm) due to the low temperature. Likewise, CO_2_ adsorption at 0 °C and subatmospheric pressures (which correspond to a maximum relative pressure of *p*/*p*^0^ < 0.03) is linked to the ultramicroporosity and complements the information given by the N_2_ isotherms. Table 1 depicts the methodology used to determine the textural parameters of the samples.

**Table 1:** Textural Parameters Determined from the N_2_ (−196 °C) and CO_2_ (0 °C) Adsorption Isotherms

Textural parameters		
Total pore volume (PV)	*V*_p_	Amount of N_2_ adsorbed at a relative pressure of 0.99
Surface area	BET	Brunauer–Emmett–Teller equation^23^
Micropore volume	*W*_0_	N_2_ isotherms: Dubinin–Radushkevich (DR) equation assuming a density of the adsorbed phase of 0.808 cm^3^ g^–1^ and a cross-sectional area of 0.162 nm^2^;^24^ CO_2_ isotherms: Dubinin–Radushkevich (DR) equation assuming a density of the adsorbed phase of 1.023 cm^3^ g^–1^ and a cross-sectional area of 0.187 nm^2^ ^25^^25^
Micropore surface area	*S*_DR_	
Average micropore width	*L*_0_	Stoeckli–Ballerini equation^26^
Pore size distribution	PSD	Quenched solid state (QSDFT) for N_2_ isotherms, assuming cylindrical pore geometry and both nonlocal density functional theory (NLDFT) and grand canonical Monte Carlo (GCMC) model for CO_2_ isotherms, assuming slit pore geometry^27^

### Surface Chemistry

The surface chemistry, in general, and the oxygen surface functionalities of carbon materials, in particular, play a crucial role in the adsorption of H_2_O_(v)_ at low relative pressures. Thus, to determine the effect of the carbon samples’ surface chemistry on the adsorption of H_2_O_(v)_, temperature-programmed desorption (TPD) tests were carried out (the experimental protocol is described in the Supporting Information).

## Adsorption studies

The equilibrium of adsorption and the uptake rates of CO_2_ and H_2_O on the selected carbon adsorbents was determined from adsorption tests carried out under different experimental conditions typical of CO_2_ postcombustion capture (temperature of 50 °C and several CO_2_ partial pressures). The equipment and the experimental methodology are described below.

### Volumetric experiments

Adsorption isotherms of CO_2_ and H_2_O were collected at 50 °C. Single-component CO_2_ adsorption isotherms were collected in a volumetric device, TriStar 3000 from Micromeritics, where the temperature was controlled by a Thermo Haake thermostatic bath. H_2_O_(v)_ adsorption isotherms were determined in a volumetric device, VSTAR, from Anton Paar QuantaTec by the company Gas to Material Technologies S.L. Before each measurement, samples were outgassed at 100 °C under vacuum overnight.

### Gravimetric tests

The nature of adsorbents and the selected operating conditions can substantially affect the CO_2_ adsorption capacity.^28^ Despite the hydrophobic character of activated carbons, the gas separation efficiency decreases under moisture conditions. Herein, a simple thermogravimetric analysis (TGA) apparatus was adapted to render a screening methodology to evaluate the influence of water vapor on CO_2_ adsorption performances using a minimal amount of sample (∼70 mg).^29^

The CO_2_ and H_2_O capture capacities of the carbon monoliths were studied under dynamic conditions in a thermogravimetric analyzer Setaram TAG24, following the procedure of Singh et al. and Plaza et al.^28,30^ but self-adapted to assess humid conditions. Dynamic measurements were performed following the conditions listed in Table 2 for both adsorbates, H_2_O and CO_2_, at 50 °C and 101.3 kPa total pressure.

**Table 2:** Conditions of the TGA experiments to determine kinetic parameters

	Adsorption stage						
Description	*T* (°C)	*P*_tot_ (kPa)	Flow rate (cm^3^ min^–1^)	*P*_CO_2__ (kPa)	*P*_H_2_O_ (kPa)	*P*_N_2__ (kPa)	Ads time (min)
CO_2_ adsorption	50	101.3	50.0	101.3			60
N_2_/H_2_O adsorption	50	101.3	104.2		4.0	97.3	30
CO_2_/H_2_O adsorption	50	101.3	104.2	97.3	4.0		30

### Single and binary experiments (CO_2_, N_2_/H_2_O, and CO_2_/H_2_O)

**Figure 1:**
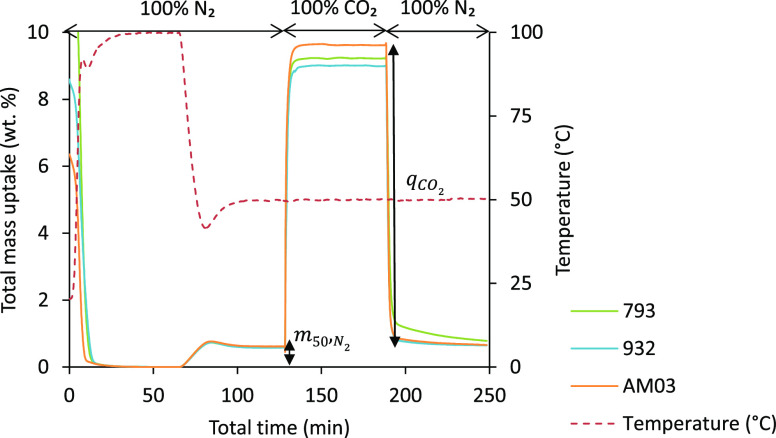
Total mass uptake vs time (full experiment) for single-component adsorption measurements on the three honeycomb carbon monoliths.

CO_2_ capture capacity experiments were performed as schematized in Figure 1: after an initial conditioning step (50 cm^3^ min^–1^ N_2_, at 100 °C for 1 h) to remove any moisture and unwanted gases, and subsequent cooling down to 50 °C in N_2_ flow and mass stabilization, the sample was exposed to a gas stream with 100 vol % CO_2_. Finally, the samples were regenerated by switching the feed gas back to 100 vol % N_2_ at a constant temperature of 50 °C.

**Figure 2:**
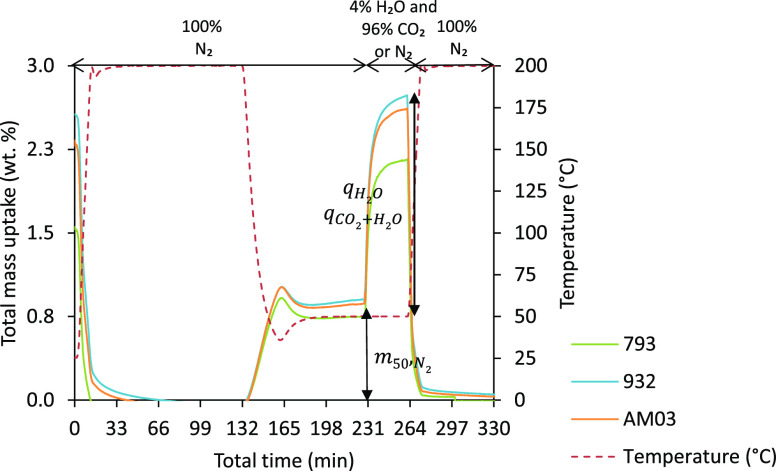
Total mass uptake vs time (full experiment) for binary adsorption measurements on the three honeycomb carbon monoliths.

Likewise, binary adsorption experiments in the presence of water vapor were performed as shown in Figure 2: after an initial conditioning step (100 cm^3^ min^–1^ N_2_, at 200 °C for 2 h), and subsequent cooling down to 50 °C in N_2_ flow and mass stabilization, the sample was exposed to a mixture consisting of 96 vol % (N_2_ or CO_2_) and 4 vol % H_2_O for half an hour. Finally, the samples were regenerated by switching the composition of the feed gas to 100 vol % N_2_ and elevating the temperature to 200 °C.

During the cooling step, the mass uptake increases due to the N_2_ adsorption. Once the temperature of the sample stabilizes, so does the mass of the sample as it reaches thermal and adsorption equilibrium with the gas phase. By running blank experiments, buoyancy and dragging effects were appropriately corrected.

During the adsorption step, the composition of the feed switched from 100% N_2_ to 100% CO_2_ in the case of the single-component adsorption experiments and to 96% (CO_2_ or N_2_) and 4% H_2_O_(v)_ for the binary experiments. The temperature kept constant at 50 °C. The mass of the sample increases due to the H_2_O_(v)_ and/or CO_2_ adsorption. The total mass uptake *q*_*i*_ is expressed in weight percentage (see Table 3) taking as a reference the mass of the sample at the end of the stabilization stage (i.e., N_2_ adsorption step), where *m*_50,N_2__ is the N_2_ mass uptake at 50 °C and *m*_50,*i*_ is the mass of the sample in the flow (CO_2_ and/or H_2_O_(v)_) at the end of the adsorption step at 50 °C.

**Table 3:** Adsorption Capacities from Single and Binary Experiments

Adsorption capacity	Equation
CO_2_, H_2_O, CO_2_ + H_2_O	

It has been confirmed that the adsorption of N_2_ at 50 °C is negligible.^31^ Therefore, the total mass uptake during the binary N_2_/H_2_O experiments, represented with *q_i_* (see Table 3), corresponds to the H_2_O adsorption capacities as determined from the H_2_O adsorption isotherms (at 50 °C and 4.0 kPa), respectively. For the binary CO_2_/H_2_O experiments, the individual contributions of both components cannot be isolated and *q_i_* accounts for the joint CO_2_ + H_2_O uptake.

### Ternary experiments (CO_2_/N_2_/H_2_O)

Flue gas from cement industry contains higher CO_2_ concentrations of approximately 14–33 vol %, compared to 12–14 vol % CO_2_ for coal-fired power plants and around 4 vol % CO_2_ for gas-fired power plants.^3,18^ Furthermore, those gas streams are saturated with water vapor concentrations ranging from 5 to 10 vol % (i.e., 100% relative humidity (RH) ≈ 12.3 vol % H_2_O at 50 °C) and the temperature of the feed is never below 40 °C.^18^ For this reason, multicomponent experiments are critical to evaluate the adsorbent performance under more realistic flue gas conditions. To that end, CO_2_ capture capacities of the honeycomb carbon monoliths were estimated in the thermogravimetric analyzer under humid postcombustion capture conditions representative of cement flue gas following the aforementioned procedure. After the initial conditioning step, a simulated flue gas stream (total flow rate of 104.2 cm^3^ min^–1^) composed of 32 vol % CO_2_, 4 vol % H_2_O, and N_2_ balance, at 50 °C and atmospheric pressure fed the thermogravimetric analyzer during the adsorption stage.

## Results and Discussion

The results reported herein point at two main research areas: (i) the study of the equilibrium of CO_2_ and H_2_O adsorption that leads to the maximum capacity of each adsorbent material in the selected scenario, (ii) the study of the kinetics of CO_2_ and H_2_O adsorption analyzing the adsorption rate. The effect that the CO_2_ concentration in the feed has on the adsorption performance will also be discussed in this section.

### Textural Characterization

**Figure 3:**
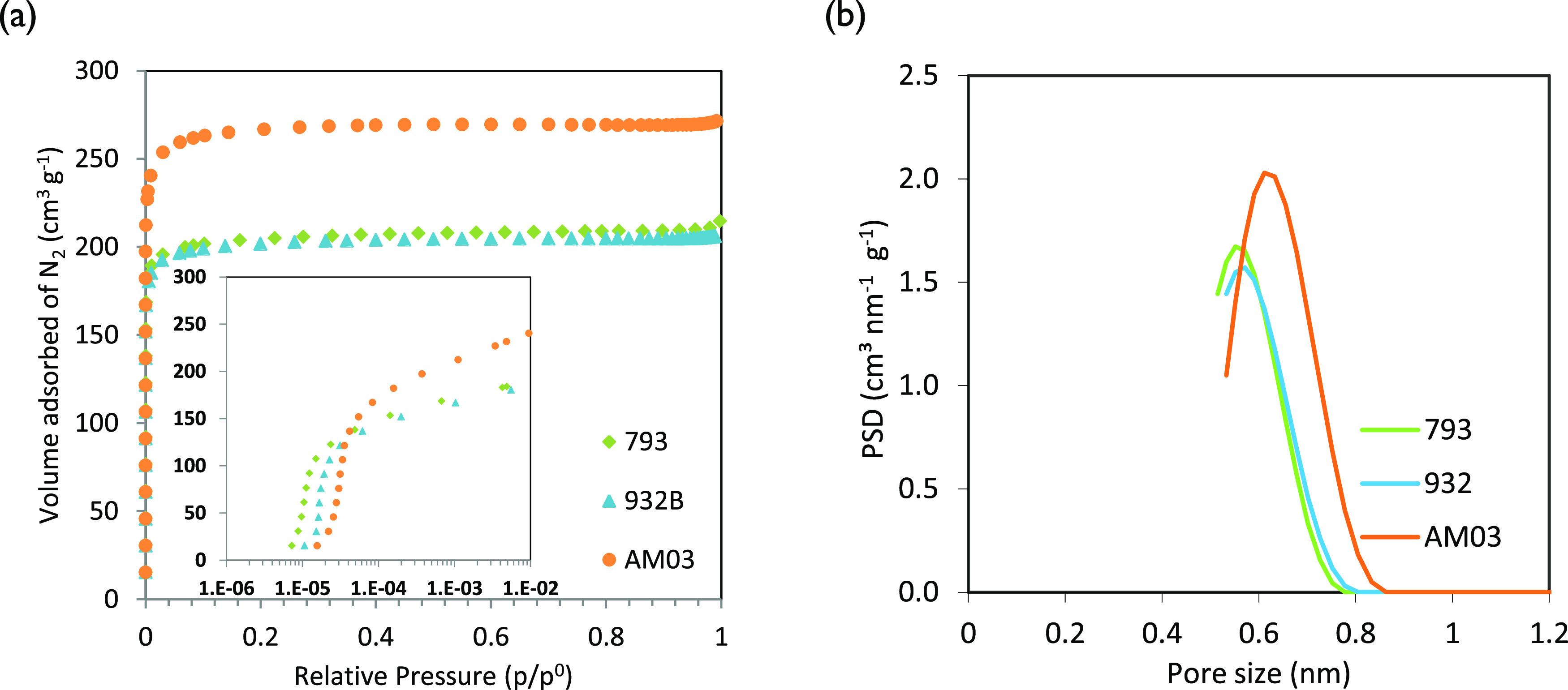
(a) N_2_ adsorption isotherms at −196 °C and (b) N_2_ adsorption QSDFT-PSD of the carbon monoliths.

The nitrogen adsorption isotherms at −196 °C and the corresponding QSDFT pore size distributions (PSD) of the three carbon monoliths are displayed in Figure 3a,b. As it can be observed, carbon monoliths present type I adsorption isotherms (IUPAC classification), characteristic of microporous structures. It has to be noticed that AM03 presents the highest adsorption of N_2_, owing to its higher microporosity development (cf. BET surface areas and pore volumes from the N_2_ adsorption isotherms shown in Table 4).

**Table 4:** Textural Parameters Estimated from the N_2_ and CO_2_ Adsorption Isotherms

	N_2_ adsorption (−196 °C)				CO_2_ adsorption (0 °C)		
Sample	*V*_p_^a^	*S*_BET_^b^	*W*_0_^a^	*L*_0_^c^	*W*_0_^a^	*L*_0_^c^	*S*_mi_^b^
793	0.33	835	0.32	0.56	0.32	0.60	1059
932	0.32	824	0.31	0.61	0.32	0.62	1024
AM03	0.42	1085	0.42	0.74	0.41	0.71	1155

a *V*, *W* in cm^3^ g^–1^.

b *S* in m^2^ g^–1^.

c *L*_0_ in nm.

The sharp knee alongside a horizontal plateau in Figure 3a shows that monoliths have narrow micropore size distributions with a limited volume of N_2_ adsorbed and the monolayer formation at low relative pressures (*p*/*p*^0^ < 0.1).^32^ It is in good concordance with the QSDFT-PSD in Figure 3b, calculated by assuming cylindrical pore geometry and adsorption branch kernel that shows single peaks centered at pore sizes <0.7 nm. This is the reason why the diffusion of N_2_ into these narrow micropores is hindered and leads to an underestimation of the micropore width (*L*_0_). It is worth noting that carbon monoliths 793 and 932 show very similar narrow PSD, while AM03 presents larger pores.

**Figure 4:**
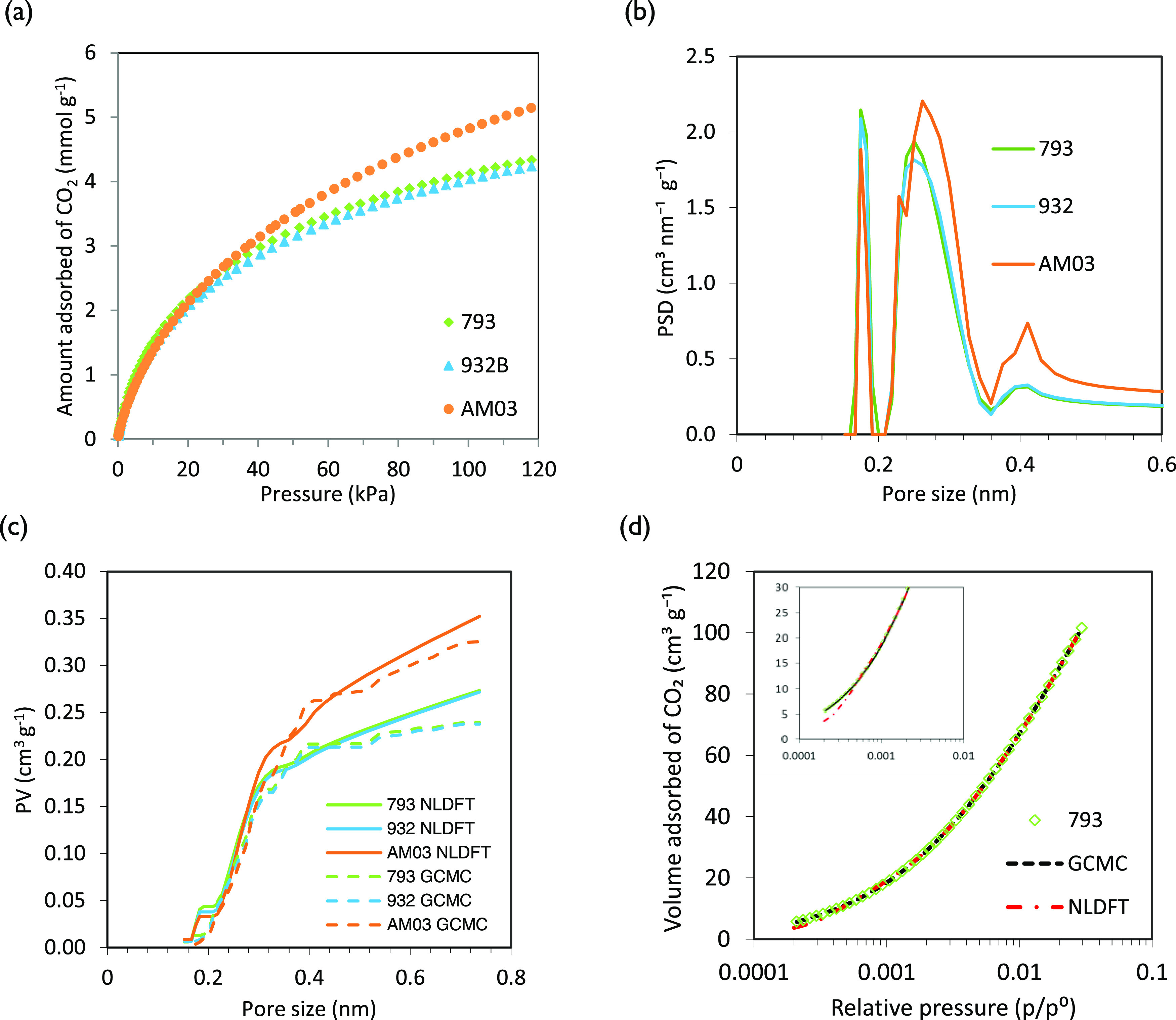
(a) CO_2_ adsorption isotherms at 0 °C, (b) CO_2_ adsorption NLDFT-PSD, (c) comparison of CO_2_ adsorption GCMC and NLDFT pore volume (PV) of the carbon monoliths, and (d) goodness of the GCMC and NLDFT model fittings.

The CO_2_ adsorption isotherms of the samples at 0 °C and the NLDFT-PSD are represented in Figure 4a,b. The micropore ratio on each sample can be evaluated by comparing the volumes adsorbed of N_2_ and CO_2_.^31^ Likewise, Table 4 includes the CO_2_ narrow micropore volume calculated through the DR equation.

NLDFT-PSD characteristics of the carbon monoliths are alike; however, AM03 has a greater pore volume (Figure 4b). Pore sizes from 0.35 to 0.5 nm can contain one layer of CO_2_ molecules, whereas for those from 0.65 and 0.8 nm, the adsorbate experiences a change to a two-layer structure. It is interesting to note that differential NLDFT-PSD exhibits a minimum at ca. 0.2 nm. The appearance of this minimum could be a consequence of the limitations of the model, which derive from the strong packing effects displayed by the parallel wall model along with the assumption of homogeneity of the surface. Figure 4c shows the application of the grand canonical Monte Carlo (GCMC) method to the CO_2_ adsorption isotherms to get further insights into the pore size distributions. This model has been customarily employed to characterize carbons assuming a simplified physical structure of microporous carbons.^33^ The combined use of these models is very useful to improve the outcome and complement the results. However, the main drawback of both sets of equations in the models is the assumption of a structureless, chemical, and geometrically smooth surface model.^34,35^ The assumption of slit-shaped pores and the associated shortcomings frequently pose disagreements in the pore size distributions extracted from adsorption isotherms. It becomes more evident in the networking and molecular sieving effects, as well as in the specific interactions between adsorbate and carbon.^33,34,36^

Contrary to the trend observed for other activated carbons in which both distributions (NLDFT and GCMC) in the ultramicroporous region (width <0.7 nm) were very comparable,^37^ for the carbon monoliths under study, the GCMC CO_2_ isotherms generated at subatmospheric pressures (Figure 4c) provide more reliable information about the PSD of the samples than the NLDFT model (Figure 4b). The better fitting of GCMC is illustrated in Figure 4d for monolith 793.

The fit of the GCMC model is particularly good for the narrower pore widths where the NLDFT presents the aforementioned gap or artifact. Deviations between both models are particularly significant in pore sizes between 0.65 and 0.8 nm where the adsorbate experiences a change from a one- to two-layer structure. In this pore range, spherical molecules form dense packing but the three-center CO_2_ molecules form a less-dense structure due to a trade-off between the tendency to lie flat to the wall and the tendency to form T-like configurations due to the quadrupole.^38^ Overall, GCMC deduced that most pore volume concentrates at sizes below 0.525 nm, therefore in the narrow micropores. Besides, about 43–48% of the total narrow micropore volume is between 0.325 and 0.425 nm. As we have explained elsewhere,^39^ a tailored porous network with ca. 40–46% of ultramicropores of less than 0.5 nm and an irrelevant presence of pores >0.7 nm allows a 40% increase in the CO_2_ retention capability for materials with similar micropore volume.

It is interesting to note that a key factor of the carbon dioxide adsorption is the average micropore width (*L*_0_): lower values of *L*_0_ give stronger adsorption potentials that can enhance the filling of the narrower microporosity with the CO_2_ molecules. Therefore, activated carbons with small average micropore widths alongside good microporosity development may be great candidates to CO_2_ capture.^21,39^

As can be appreciated in Table 4, AM03 displays the largest volume of narrow microporosity. The textural characteristics from CO_2_ adsorption follow the same pattern observed in N_2_ adsorption. From the CO_2_ adsorption isotherms, the values obtained for *W*_0_ and *L*_0_ are within the typical ranges described for activated carbons and point out a good development of the narrow microporosity in the carbon monoliths.^20,40^

The microporosity features have a strong influence on the CO_2_ uptake but also influence water vapor adsorption. The surface oxygen functional groups content promotes water vapor uptake at low pressures, but from medium to high pressures, the micropore filling is responsible for the adsorption capacity of the activated carbon. Moreover, the size of the formed water clusters is dependent on the micropore width.^41,42^

Hence, the narrow range of microporosity present on the carbon monoliths allows us to selectively adsorb CO_2_ at low partial pressures.^31,43^ Among the evaluated monoliths, the more developed micropore network in AM03 suggests that this carbon monolith may exhibit both the highest CO_2_ and H_2_O adsorption capacities.

### Surface Oxygen Functional Groups

The monitoring of labile surface oxygen groups in the carbons in the form of CO and CO_2_ as a function of temperature was followed through TPD tests (cf. Figure S1 in the Supporting Information). Integration and deconvolution of these curves rendered the concentration of oxygen surface functionalities, which are shown in Table 5. It can be seen in this table that the number of oxygen functionalities that evolve as CO is much higher than those that give CO_2_. The total amount of oxygen functionalities on the surface of carbons is given by the sum of CO + CO_2_, as indicated in Table 5.

**Table 5:** Amount of CO and CO_2_ Evolved during the TPD Experiments

Sample	CO (μmol g^–1^)	CO_2_ (μmol g^–1^)	CO/CO_2_	CO + CO_2_ (μmol g^–1^)
793	1515	567	2.7	2082
932	1775	665	2.7	2440
AM03	2402	796	3.0	3198

Overall, honeycomb carbon monoliths present a basic surface as explained by the higher content of surface functionalities that decompose into CO. Moreover, all samples show similar oxygen functionality ratios on their surfaces, as can be inferred from the values of CO/CO_2_. Only carbon monolith AM03 shows a slightly acidic surface. Besides, the surface oxygen functionalities amount (CO + CO_2_) is analogous to other ACs.^20^

Tables 6 and 7 display the distribution of the main oxygen functionalities (i.e., carbonyl and quinone, pyrone and chromene, together with carboxylic, peroxide, and lactone), which were estimated from the deconvolution of the CO and CO_2_ profiles.

**Table 6:** Distribution of Oxygen SurfaceComplexes Estimated from CO-TPD Profiles

Sample	Carbonyl and quinone (μmol g^–1^)	Pyrone and chromene (μmol g^–1^)
793	1083	373
932	1389	335
AM03	2349	337

**Table 7:** Distribution of Oxygen Surface Complexes Estimated from CO_2_-TPD Profiles

Sample	Carboxylic (μmol g^–1^)	Peroxide (μmol g^–1^)	Lactone (μmol g^–1^)
793	272	131	165
932	289	200	177
AM03	292	232	273

The deconvolution of the TPD profiles clearly shows the similarities of the honeycomb carbon monoliths surfaces in terms of oxygen surface functionalities development, the slight differences ascribed to the intensity of the activation conditions. As can be observed in Table 6 and 7, all of the samples present similar contents of pyrone and chromene while sample AM03 doubles the content of carbonyl and quinone and also exceeds in peroxide and lactone.

### Volumetric CO_2_ and H_2_O_(v)_ Adsorption

**Figure 5:**
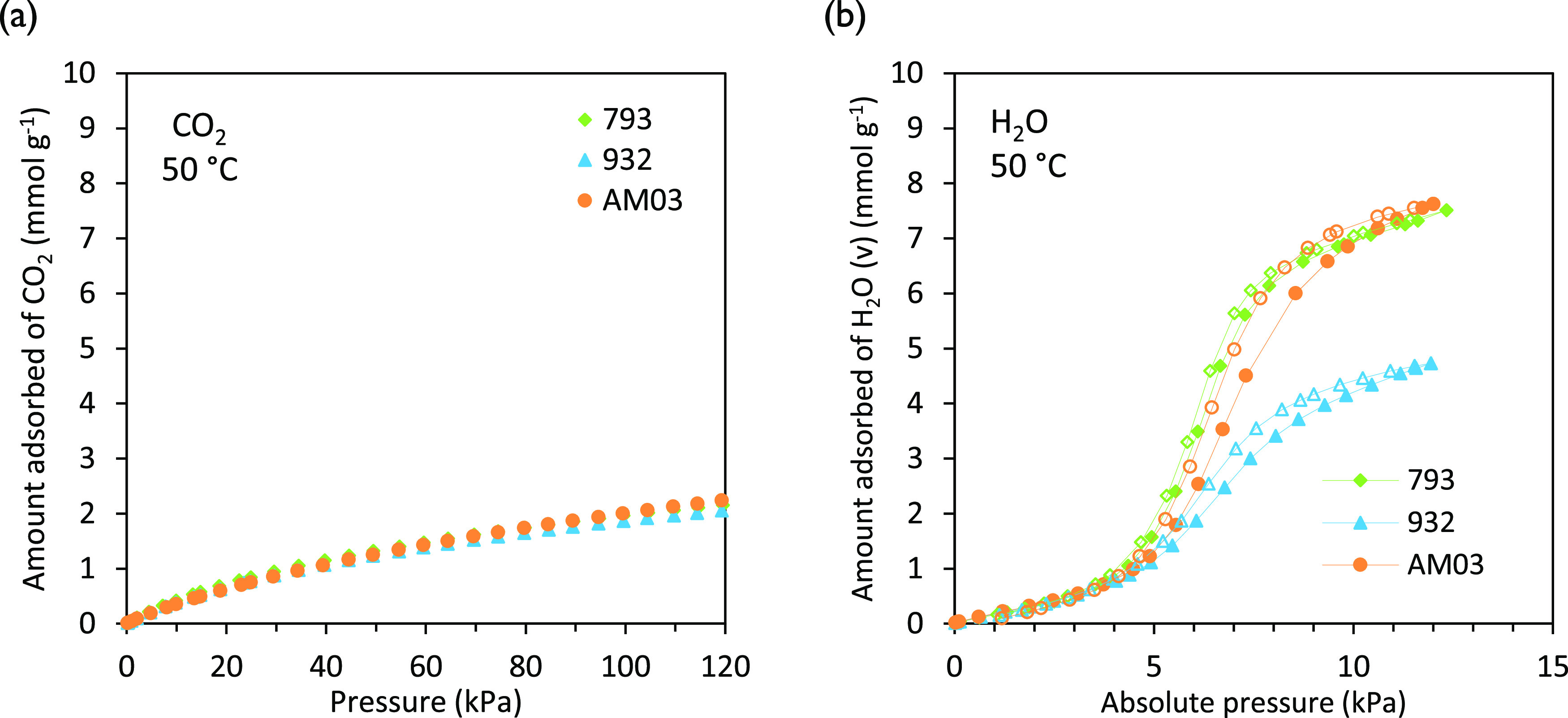
Equilibrium adsorption isotherms of (a) CO_2_ and (b) H_2_O_(v)_ at 50 °C on the honeycomb carbon monoliths 793, 932, and AM03.

Water vapor is an unavoidable flue gas component and competes with CO_2_ for the adsorption on a solid sorbent surface.^18,20^ Therefore, it is important to evaluate the capacity of carbon to adsorb CO_2_ and H_2_O_(v)_, when addressing CO_2_ capture from industrial flue gases. The CO_2_ and H_2_O adsorption isotherms of the three carbon monoliths at 50 °C are presented in Figure 5. At a given pressure, all of the carbons adsorb a greater amount of H_2_O_(v)_ than CO_2_. Temperature limits the pressure range for water vapor adsorption due to condensation (see Figure 5b) given that the vapor pressure (*p*^0^) of water at 50 °C is 12.3 kPa. Differences in adsorption uptakes between the three monoliths are however more relevant for H_2_O_(v)_.

Globally, the CO_2_ adsorption isotherms belong to type I (IUPAC classification), representative of stronger adsorbate–adsorbent interactions. CO_2_ uptakes are similar for the three monoliths in the lower-pressure range that corresponds to CO_2_ postcombustion capture conditions (i.e., up to ∼40 kPa) and slightly differ at higher pressures. This is due to the similarities observed in the textural development of the three carbon monoliths in the narrow microporosity range where CO_2_ adsorption is likely to occur by a micropore filling mechanism.

Likewise, water vapor adsorption on the carbon monoliths displays the typical type V topology (IUPAC classification). It is characterized by small uptakes at low pressures (absolute pressures below 4 kPa in Figure 5b) and a hysteresis loop that covers most of the pressure range. All of the carbon monoliths, 793, 932, and AM03, exhibit similar water vapor adsorption capacities at low pressures (∼3 kPa), wherein the amount of H_2_O_(v)_ adsorbed on the carbon correlates with the number of oxygen groups present on the activated carbon surface. At higher pressures, from around 3.8 kPa for 793 and AM03 up to 4.0 kPa for 932, water–water interactions predominate and a sharp rise of the isotherm occurs due to the water cluster growth around primary adsorption centers and the micropore volume filling.^44^ In the third region of the H_2_O_(v)_ isotherm of the carbon monoliths, at pressures above 8.5 kPa (cf. Figure 5b), a wide micropore filling takes place. Significantly higher uptakes are attained at saturation for samples 793 and AM03 compared to 932.

The characteristics of the carbon material such as pore shape and connectivity and the surface chemistry affect the position, extension, and width of the water hysteresis loop.^45,46^ Carbon monolith AM03, with a wider micropore size distribution, shows pronounced hysteresis because the water molecules adsorb in large clusters that then enter the micropore volume and finally desorb through uniform molecular evaporation.^32^

The differences observed in the adsorption performance of the honeycomb carbon monoliths were analyzed in terms of the equilibrium separation factor, assuming simulated cement flue gas after desulphurization, at 50 °C and 101.3 kPa, with two compositions (vol %): 32% CO_2_ and 4% H_2_O_(v)_, that will be evaluated hereafter experimentally, and other composition with a higher water vapor content (10% H_2_O_(v)_) (see Table 8). Since the small pores are the preferred adsorption sites for the molecules of CO_2_ and H_2_O, both adsorbates show strong competition for the adsorption on these sites at such low partial pressures.^47^

**Table 8:** H_2_O/CO_2_ Separation Factor for a Simulated Cement Flue Gas Previously Desulfurized (32% CO_2_ and 4 and 10% H_2_O) at 50 °C and 101.3 kPa

	aSeparation factor	

Sample	4% H_2_O	10% H_2_O
793	7	22
932	7	15
AM03	7	24

a

There is a prevalence of H_2_O adsorption over CO_2_, as estimated by the separation factor, as defined in Table 8. However, the values in Table 8 are significantly lower than those previously reported in the literature for carbon monoliths, 34 and 89, under postcombustion capture conditions in a coal-fired power plant.^22^

### CO_2_ Adsorption Measurements: Thermogravimetric Tests

#### CO_2_ and H_2_O Adsorption

**Figure 6:**
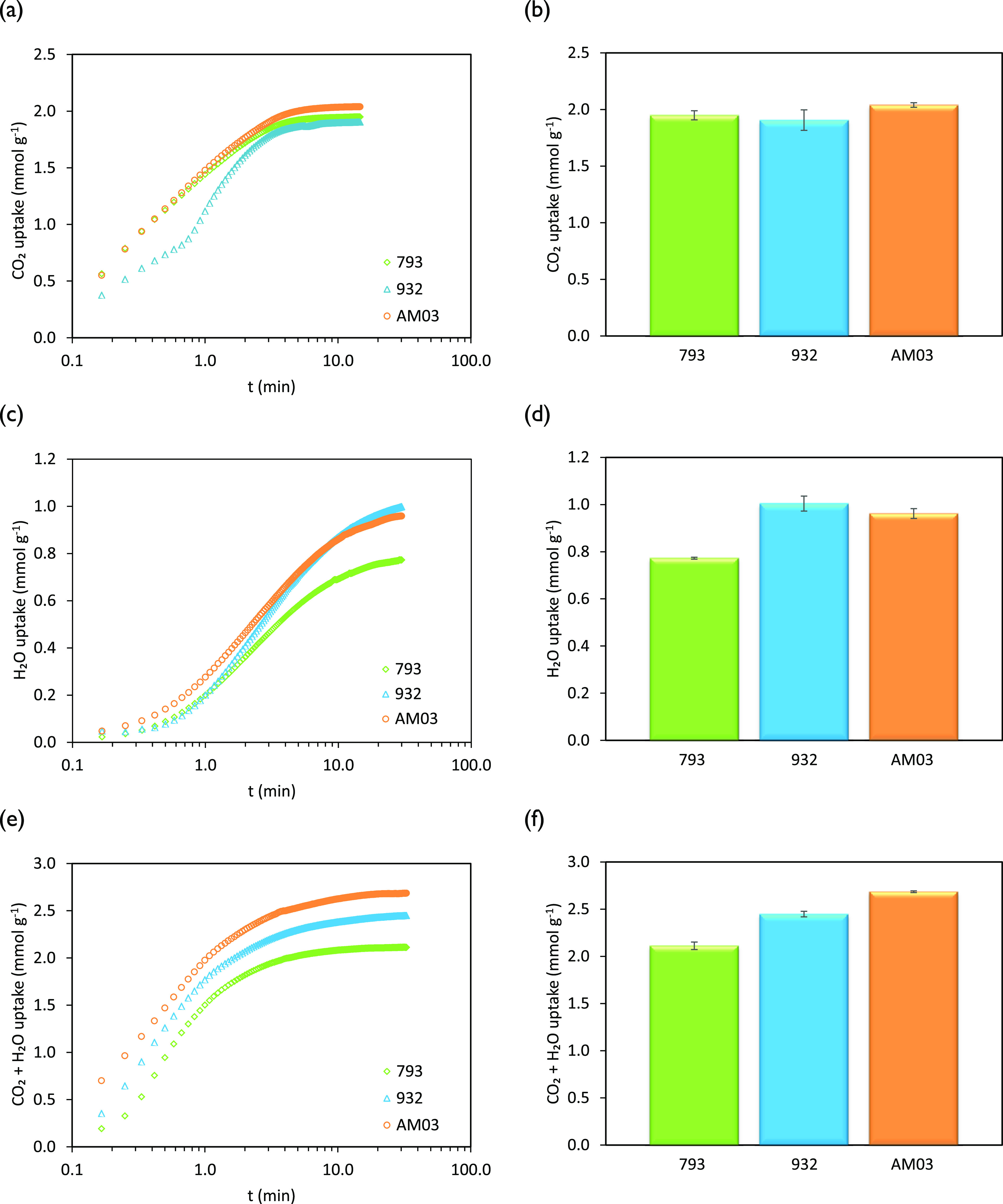
CO_2_, H_2_O, and CO_2_ + H_2_O uptakes of the honeycomb carbon monoliths at 50 °C under (a, b) pure CO_2_ flow; (c, d) 4.0 vol % H_2_O_(v)_, N_2_ balance; and (e, f) 4.0 vol % H_2_O_(v)_, CO_2_ balance. Plots on the left-hand side show the uptake evolution with time. Plots on the right-hand side show the maximum uptake at equilibrium.

Figure 6a,b shows TGA results using small pieces of honeycomb carbon monoliths and operating the adsorption step at 50 °C in 100% CO_2_ flow. The three samples exhibited very fast adsorption in the first few minutes where the major CO_2_ uptake takes place (ca. 1.7 mmol CO_2_ g^–1^ after 2 min that corresponds to approximately 86% of the equilibrium capacity). Then, the uptake continued to increase at a slower pace and reached a plateau within 6 min (see Figure 6a). The amount adsorbed at equilibrium represents the maximum adsorptive sample capacity. We have verified that the CO_2_ mass uptake in this type of single-component gas adsorption measurements is in good concordance with the CO_2_ adsorption capacities determined from the adsorption isotherms of CO_2_ at 50 °C up to 101.3 kPa.^6,31^ It is evidenced comparing the data reported in Figures 5a and 6b.

Analyzing the textural characteristics of the carbon monoliths (Table 4) and the CO_2_ adsorption capacities (Figure 6a,b), it follows that the differences in adsorption uptake between the carbon monoliths are in agreement with their microporosity developments.

The CO_2_ adsorption capacities of the carbon monoliths evaluated in this study surpass the values reported in the literature for carbon monoliths at 50 °C and 100% CO_2_ and even those achieved at more favorable conditions (25–35 °C, 100% CO_2_).^21,28,43,48,49^ Among the three carbon monoliths, the maximum CO_2_ adsorption capacity was observed for AM03 (2.04 mmol CO_2_ g^–1^) due to the greater textural development, as described above. Samples 793 and 932 reached capacities slightly below 2 mmol CO_2_ g^–1^ that are still significant. The CO_2_ adsorption capacities follow the order: AM03 > 793 > 932.

To evaluate the effect of H_2_O_(v)_ on the CO_2_ capture performances of the carbon monoliths, two sets of binary adsorption experiments feeding water vapor to the system were conducted in the TGA at 50 °C and atmospheric pressure: (i) feeding 4.0 vol % H_2_O_(v)_, N_2_ balance, and (ii) feeding 4.0 vol % H_2_O_(v)_, CO_2_ balance. The H_2_O_(v)_ uptakes during the binary N_2_/H_2_O_(v)_ adsorption experiments are summarized in Figure 6c,d, while the global uptakes during the binary CO_2_/H_2_O_(v)_ adsorption experiments are presented in Figure 6e,f.

As can be observed in Figure 6c, the H_2_O profiles took longer times to attain the equilibrium capacity compared to the single-component CO_2_ adsorption experiments. This difference in time is due to significantly slower kinetics of adsorption of H_2_O that require approximately 25 min to reach the plateau characteristic of the equilibrium (H_2_O uptake within ∼9 min reached 0.7–0.8 mmol g^–1^, 87% of the maximum uptake). The amount of water adsorbed at equilibrium represents the maximum capacity of adsorption of the sample. We have verified that the H_2_O mass uptakes during the binary N_2_/H_2_O adsorption experiments (Figure 6d) correspond to the H_2_O adsorption capacities as determined from the adsorption isotherms of H_2_O_(v)_ at 50°C and 4.0 kPa. Therefore, the adsorption of N_2_ under these conditions is negligible.

In Figure 6d, it is observed that carbon monoliths 932 and AM03 displayed comparable H_2_O profiles. Sample 932 reached a maximum uptake of approximately 1.0 mmol g^–1^. Despite the similarities in the narrow microporosity of these samples, 793 showed a much lower H_2_O adsorption capacity. The aforementioned surface oxygen functional groups content on each sample would account for the differences in water vapor uptake at low pressures.^20^ Regarding the H_2_O adsorption capacity, the adsorbents follow the order: 932 > AM03 > 793.

When a binary mixture of CO_2_/H_2_O fed the TGA, the combined CO_2_ + H_2_O (Figure 6f) capacity of the adsorbents increased for samples 932 and AM03 compared to the CO_2_ uptake during the single-component experiments. The profiles show a steep uptake in the first few minutes, similar to the single-component experiments in pure CO_2_, which indicate fast adsorption kinetics. Most of the maximum uptake (86%) is reached within ∼2 min, but the presence of water vapor seems to slow down the achievement of the plateau to 10 min (see Figure 6e). We have demonstrated in previous work that low-humidity conditions, like those studied in this work, do not alter the CO_2_ capture performance of the adsorbents.^50^ Additional shreds of evidence are presented in the Supporting Information. Thus, assuming that the slower adsorption kinetics of H_2_O do not hinder the faster adsorption of CO_2_ at the beginning of the experiment, the carbon monoliths may reach the equilibrium CO_2_ uptakes at the evaluated conditions (50 °C and 97.3 kPa CO_2_). These maximum CO_2_ uptakes were obtained from the adsorption isotherms of CO_2_ at 50 °C (see Figure 5a). Therefore, the excess uptake, once the plateau is reached, might be attributed to H_2_O adsorption. Table 9 summarizes the CO_2_ and H_2_O adsorption capacities during the binary CO_2_/H_2_O adsorption experiments.

**Table 9:** CO_2_ and H_2_O Adsorption Capacities of the Carbon Monoliths Assessed from the Binary CO_2_/H_2_O Adsorption Experiments at 50 °C under 4.0 vol % H_2_O_(v)_, CO_2_ Balance

Sample	CO_2_ adsorption capacity (mmol g^–1^)	H_2_O adsorption capacity (mmol g^–1^)
793	1.9	0.2
932	1.8	0.6
AM03	2.0	0.7

During the two sets of binary experiments, the H_2_O_(v)_ concentration in the feed gas remained unchanged (4 vol % ), but it must be noted that the water vapor uptake significantly reduced in the presence of CO_2_. In the binary CO_2_/H_2_O experiments, there is competitive adsorption between the two strong adsorbates. However, the faster kinetics of CO_2_ alongside the surface oxygen functional groups content and microporosity development characteristic of each sample might limit the water vapor adsorption in the presence of CO_2_. Given that the relative pressure of water vapor in the isotherms correlates to the relative humidity (RH), it is expected that the equilibrium water vapor uptake under the experiment conditions matches the theoretical 33% RH of the feed. However, the relative humidity corresponding to the calculated H_2_O uptakes from the H_2_O adsorption isotherms at 50 °C render values below the theoretical one: 8, 29, and 31% for 793, 932, and AM03, respectively. It would confirm that the adsorption of H_2_O does not reach equilibrium in the presence of CO_2_ under the evaluated conditions and the characteristics of sample 793 substantiate the effect. Consequently, in terms of CO_2_ + H_2_O adsorption capacity, the adsorbents follow the order: AM03 > 932 > 793. The H_2_O adsorption capacity of each sample is the key factor defining the final combined uptake and relegates sample 793 to the last position (see Table 9).

#### CO_2_ Adsorption under Flue Gas Conditions

The performance of carbon monoliths was evaluated at partial pressures relevant to flue gas emissions from the cement industry. Therefore, multicomponent adsorption experiments were carried out to evaluate the performance in a simulated flue gas stream (total flow rate of 104.2 cm^3^ min^–1^) composed of 32 vol % CO_2_, 4 vol % H_2_O, and N_2_ balance, at 50 °C and atmospheric pressure.

**Figure 7:**
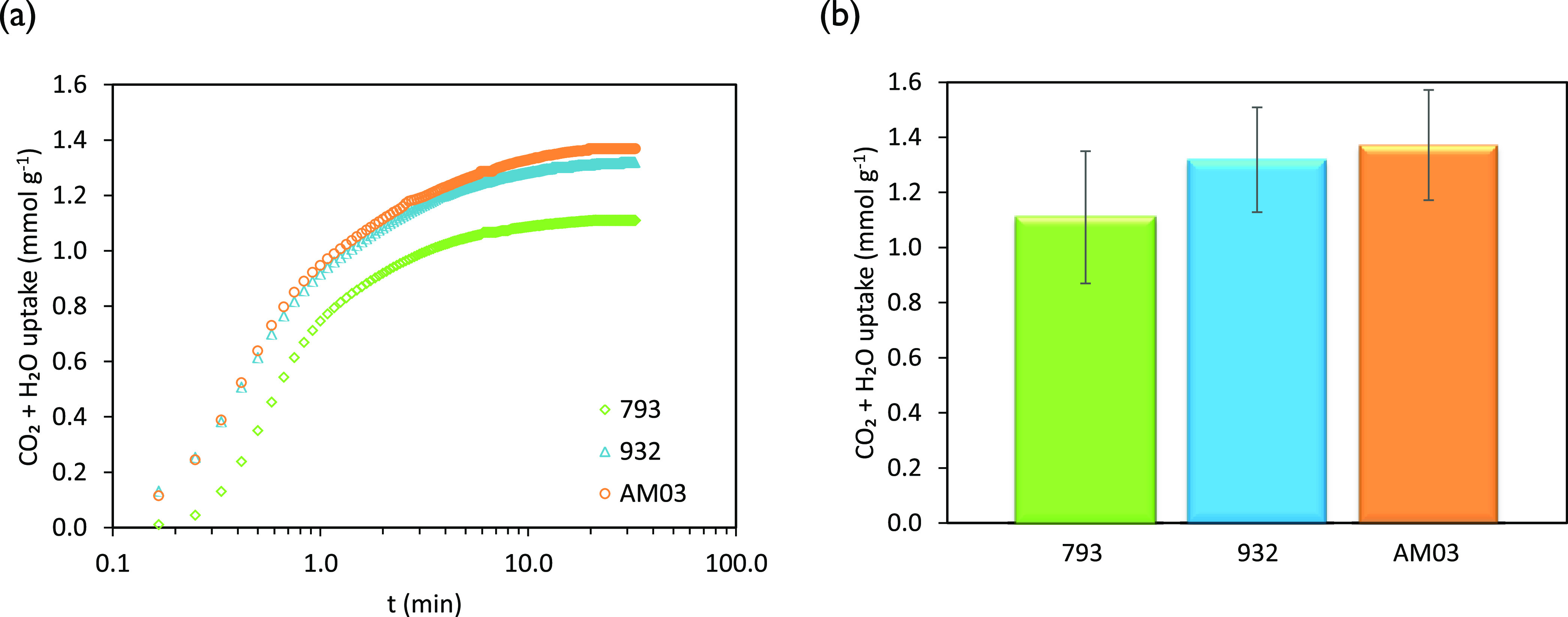
CO_2_ + H_2_O uptakes of the honeycomb carbon monoliths under simulated cement flue gas conditions at 50 °C: (a) evolution of the uptake with time and (b) maximum uptake at equilibrium.

**Figure 8:**
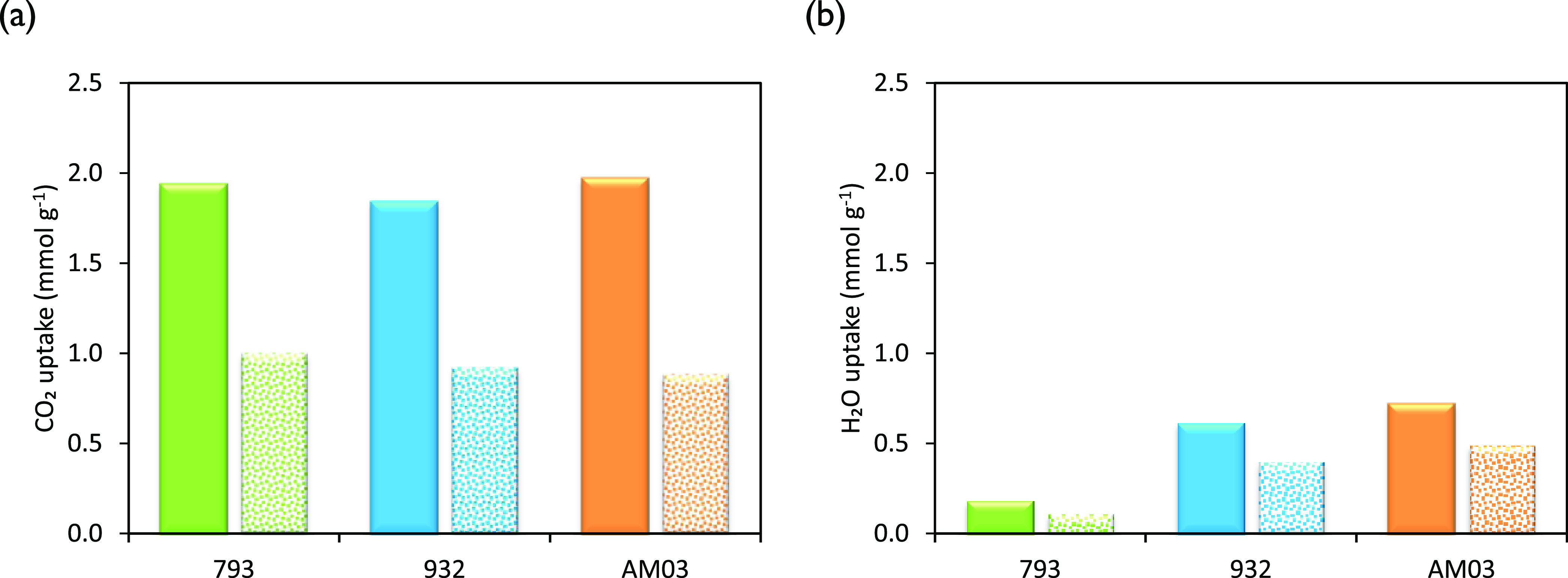
Comparison of the experimental values of the CO_2_ and H_2_O uptakes at two CO_2_ partial pressures (filled bars: 97.3 kPa CO_2_ and 4.0 kPa H_2_O; dotted bars: 32.1 kPa CO_2_, 4.0 kPa H_2_O, balanceN_2_ balance).

As can be deduced from Figure 7a, the profiles of the CO_2_ + H_2_O uptake (N_2_ adsorption is considered negligible under these conditions, as confirmed in a previous study^22^) for the carbon monoliths in the presence of 32 vol % CO_2_ show similarities to those of the binary experiments in 96 vol % CO_2_, although the amounts adsorbed in absolute terms significantly reduced given the lower CO_2_ partial pressure. The total mass uptake (Figure 7b) was calculated following the criteria discussed for the CO_2_/H_2_O binary experiments. The excess with regard to the equilibrium CO_2_ uptake under the evaluated conditions was attributed to H_2_O adsorption. It has to be noticed that the relative contribution of the water uptake to the combined CO_2_ + H_2_O uptake under simulated flue gas conditions of the cement industry is more relevant than in the CO_2_/H_2_O binary experiments (see Figure 8a,b). For instance, the H_2_O uptake for sample AM03 in binary experiments accounted for 27% of the total uptake, while this percentage increased to 36% in ternary experiments.

Table 10 summarizes the isolated CO_2_ and H_2_O contributions determined from the multicomponent adsorption experiments in simulated flue gas. Compared to the binary experiments, the CO_2_ uptake experienced a substantial reduction (see Figure 8a), in agreement with the reduced CO_2_ partial pressure in the feed that decreases the driving force for CO_2_ adsorption.^28^ Thus, a stronger competition in adsorption between CO_2_ and H_2_O is observed (see Figure 8b) that in turn reduces the combined adsorption capacity below the values obtained in pure CO_2_ and binary CO_2_/H_2_O adsorption experiments.

**Table 10:** CO_2_ and H_2_O Adsorption Capacities of the Carbon Monoliths Assessed from the Experiments in Simulated Cement-Industry Flue Gas (32 vol % CO_2_, 4 vol % H_2_O, andN_2_ Balance, at 50 °C and Atmospheric Pressure)

Sample	CO_2_ adsorption capacity (mmol g^–1^)	H_2_O adsorption capacity (mmol g^–1^)	CO_2_/H_2_O ratio
793	1.0	0.1	10
932	0.9	0.4	2
AM03	0.9	0.5	2

Thus, in terms of CO_2_ + H_2_O adsorption capacity in the flue gas stream, the adsorbents follow the order: AM03 > 932 > 793. However, besides the combined uptake, it is important to highlight the ability of the adsorbents to selectively separate CO_2_ from the other components in the flue gas. Under conditions relevant to cement-industry flue gas, sample 793 showed the highest CO_2_ uptake in conjunction with the lowest H_2_O uptake, which translates into the highest ratio CO_2_/H_2_O. Thus, sample 793 seems the best carbon monolith candidate for capturing CO_2_ from cement-industry flue gas streams under the evaluated conditions.

### Adsorption Kinetics

In porous adsorbents, mass or heat transfer resistances mostly control the overall rate of the adsorption/desorption process because physical adsorption at the active surface takes place very rapidly, and thus the intrinsic rate of sorption is not the rate-controlling step.^51^ High-capacity CO_2_ adsorbents present extended narrow microporosity. Transport through these pores takes place principally by diffusion that could also control the process overall rate. The development of adsorption processes together with their proper design and optimization needs a comprehensive understanding of the intricacies of diffusion behavior in porous materials.

For practical applications, it is crucial to comprehend the dynamic behavior of the adsorption system.^52^ The adsorption kinetics analysis determines the residence time and the rate-controlling mechanism of the process. It is a requirement to define the fixed-bed performance or any other flow-through process. High capacity and slow kinetics of adsorption or the combination of low capacity and fast adsorption kinetics might not be suitable for the selected application.^53,54^ Therefore, in any adsorption application, it is essential to establish the diffusional behavior and the macroscopic adsorption/desorption kinetics relationship through mathematical models. The adsorption data obtained during the thermogravimetric tests were fitted to the four models summarized in Table 11. The use of detailed mechanistic models in industrial-plant simulations is not appropriate due to their intrinsic computational load. More simplistic relations, such as those in Table 11, that can be readily solved are preferred.^52^ Thus, the four kinetic models may contribute to gaining more insights into the water vapor effects on the CO_2_ adsorption kinetics on the carbon monoliths.

**Table 11:** Adsorption Kinetics Empirical Models

Kinetic model	Equation	Ref.
Pseudo-first-order	*q_t_* = *q*_e_(1 – e^–*k*_f_*t*^)	(6)
Avrami	*q_t_* = *q*_e_(1 – e^(−(*k*_A_*t*)*n*_A_)^)	(6)
Fractional-order		(55)
Exponential decay-2	*q_t_* = *q*_e_ + *a*_1_ e^–*t*/*b*_1_^ + *a*_2_ e^–*t*/*b*_2_^	(13)

Table 11 lists the equations associated with these kinetic models, where *t* is the time elapsed from the beginning of the adsorption stage, *q*_*t*_ (mmol g^–1^) is the amount adsorbed at a given time, *q*_e_ (mmol g^–1^) represents the amount adsorbed at equilibrium, *k*_f_ (min^–1^) is the pseudo-first-order rate constant, *k*_A_ (min^–1^) and *n*_A_ are the Avrami kinetic constant and exponent, respectively, *k*_*n*_ (min^–1^) is the fractional-order kinetic constant, *n* and *m* are the fractional-order model constants, and *a*_1_ (mmol g^–1^), *a*_2_ (mmol g^–1^), *b*_1_ (min), and *b*_2_ (min) are fitting parameters.

Based on the standard deviation to quantify the measured data and the predictions discrepancy from the model, the sum of squared errors (SSE) and the coefficient of determination (*R*^2^) were estimated. And the equations are shown in Table 12, where *q*_*t*,exp_ and *q*_*t*,pred_ are the experimentally measured and model-predicted adsorption capacities, respectively; *N* is the number of experimental data points for each sample; and *p* is the number of parameters of the model.

**Table 12:** Goodness of Fitting of the Kinetic Models

Sum of squared errors (SSE)		(6)
Coefficient of determination (*R*^2^)		(6)

Hereafter, the fittings of the honeycomb monolith 793 data will be shown in the figures for illustrative purposes.

#### Adsorption Rate

**Figure 9:**
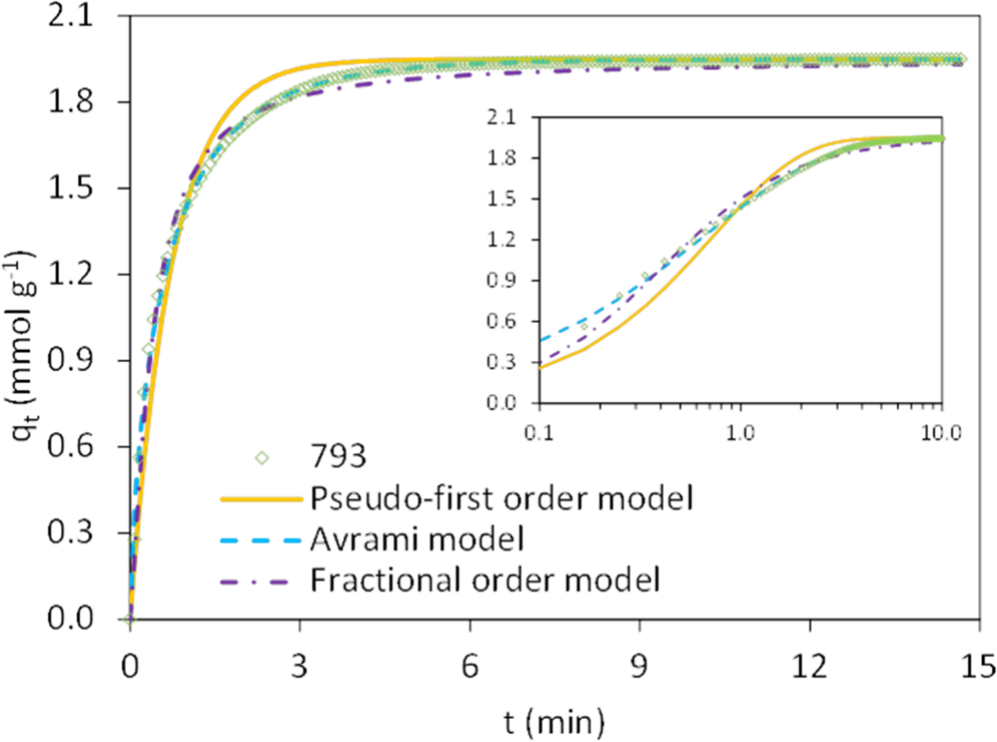
Fittings for the adsorption of pure CO_2_ at 50 °C on carbon monolith 793 (open symbols, experimental data; solid lines, pseudo-first-order model; dashed lines, Avrami’s model; and dotted lines, fractional-order model).

First, the kinetics of CO_2_ adsorption in pure CO_2_ atmosphere were evaluated by the pseudo-first-order, Avrami, and fractional-order models (see Figure 9). Carbon monoliths show two-stage adsorption that corresponds to mass transfer resistance^56,57^ and to proper surface adsorption, which is generally very quick.^57,58^ It can be seen that the pseudo-first-order model does not suitably fit the experimental data. It underestimates the CO_2_ uptakes at the initial stages of the adsorption step but overestimates the uptakes when approaching the maximum capacity (equilibrium). This model better suits adsorption processes controlled by surface diffusion.

Avrami and fractional-order models better describe the experimental data over the entire adsorption step, indicating that CO_2_ adsorption on carbon monoliths is a complex multipath process.^59,60^ On the one hand, Avrami’s model has described the kinetics of crystallization, and it accounts for the random nucleation and subsequent growth, whereas the fractional-order model can explain different adsorption pathways, including surface and intraparticle diffusion, and interaction with active sites on the adsorbent surface (physical and chemical). Thus, the parameter *k*_n_ lumps adsorption-related factors in an overall parameter.^61,62^

The kinetic parameters values determined for the three models, the corresponding correlation coefficients (*R*^2^), and the associated sum of squared errors (SSE (%)) are presented in Table 13.

**Table 13:** Values of the Kinetic Model Parameters for the Adsorption Experiments in Pure CO_2_ at 50 °C and Atmospheric Pressure

	Pseudo-first-order			Avrami				Fractional-order				
Sample	*k*_f_ (min^–1^)	SSE (%)	*R*^2^	*k*_A_ (min^–1^)	*n*_A_	SSE (%)	*R*^2^	*k*_n_ (min^–1^)	*n*	*m*	SSE (%)	*R*^2^
793	1.36	0.05	0.966	1.51	0.71	0.01	0.998	1.36	2.65	1.30	0.03	0.989
932	0.91	0.02	0.995	0.93	0.93	0.02	0.996	0.81	2.84	1.62	0.04	0.981
AM03	1.26	0.06	0.964	1.39	0.70	0.01	0.998	1.20	2.58	1.28	0.03	0.990

The CO_2_ adsorption approximates the crystal nucleation and growth, which begins from a point and then extends to the surroundings. The values of Avrami’s exponent (*n*_A_), as can be seen from Table 13, are around 2/3 for samples 793 and AM03, and around 1 for sample 932. These reaction orders respond to the different performances observed for 932 in the first minutes of adsorption (see Figure 6a). The fractional-order model parameter *n* reflects the strong effect of the adsorption driving force (values larger than 2). Besides, *m* refers to diffusion resistance, attaining a higher value for 932 that suggests slightly fast adsorption for this carbon monolith.

Among the two models, Avrami provides the best description of the CO_2_ adsorption behavior on the honeycomb carbon monoliths, according to the values of *R*^2^ and SSE. This is in accordance with other studies on the CO_2_ adsorption kinetics on biomass-based activated carbons.^6,63^

**Figure 10:**
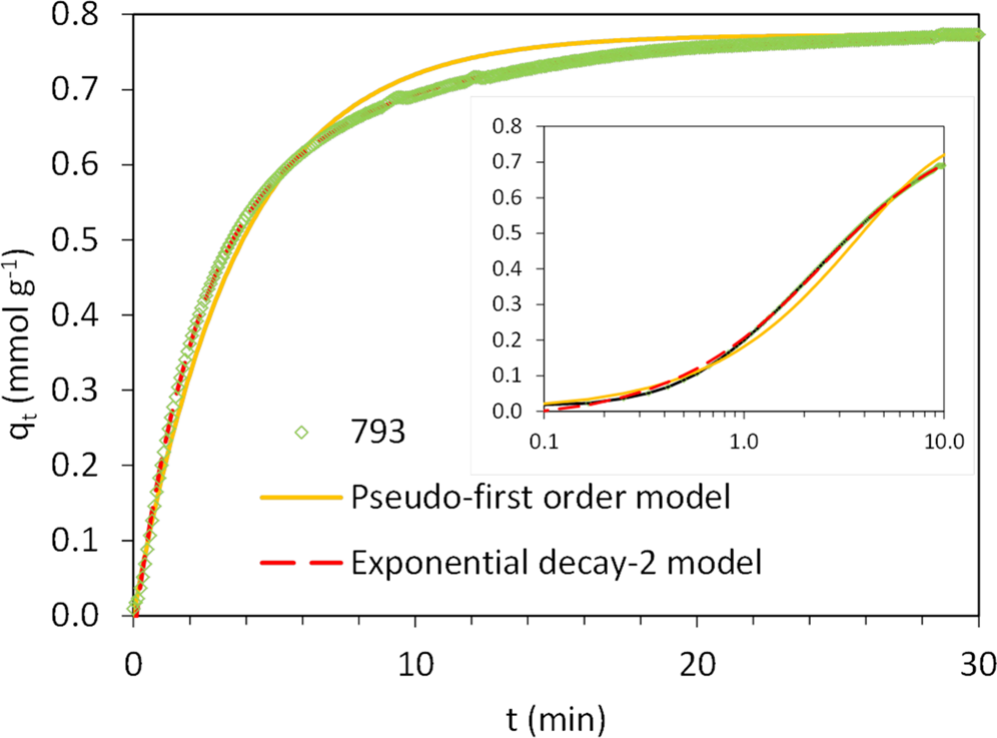
Fittings for the H_2_O adsorption at 50 °C on the carbon monolith 793 when feeding a binary mixture of N_2_/H_2_O (open symbols, experimental data; solid lines, pseudo-first-order model; and red dashed lines exponential decay-2 model).

The adsorption of water vapor on carbon monoliths has inherent complexity. It entails the water molecules and clusters diffusion in the micropore network. This diffusion occurs into those micropores that present a width smaller than the free path of the gas molecules. The kinetics of H_2_O adsorption have been evaluated with the pseudo-first-order model alongside the exponential decay-2 model. The latter have proved feasible when other kinetic models failed to represent the water vapor adsorption kinetics along the whole adsorption step.^13^ The fittings of the experimental data of sample 793 by the two models are shown in Figure 10. The exponential decay-2 model fits the adsorption data of the complete experiment and displays a correlation coefficient higher than the pseudo-first-order model. Conversely, the pseudo-first-order model again underestimates or overestimates the H_2_O uptake depending on the time interval.

The kinetic parameters values calculated for the two models, the corresponding correlation coefficients (*R*^2^), and the sum of squared errors (SSE (%)) are presented in Table 14. Overall, the exponential decay-2 model gives better fitting with values of the correlation coefficient close to 1 and significantly lower values of SSE.

**Table 14:** Values of the Kinetic Model Parameters for the Adsorption Experiments in 4 vol % H_2_O and Balance N_2_ at 50 °C and Atmospheric Pressure

	Pseudo-first-order			Exponential decay-2					
Sample	*k*_f_ (min^–1^)	SSE (%)	*R*^2^	*a*_1_ (mmol g^–1^)	*a*_2_ (mmol g^–1^)	1/*b*_1_ (min^–1^)	1/*b*_2_ (min^–1^)	SSE (%)	*R*^2^
793	0.27	0.02	0.985	–0.48	–0.32	0.51	0.14	0.003	0.999
932	0.23	0.03	0.985	–0.64	–0.41	0.42	0.12	0.007	0.999
AM03	0.27	0.03	0.977	–0.57	–0.40	0.53	0.14	0.003	1.000

Water vapor adsorbs rapidly from the outset of the adsorption step, but the adsorption rate decreases with time because of the continuous reduction in the driving force^64^ as illustrated, for instance, in the exponential decay-2 model with the kinetic parameters 1/*b*_1_ and 1/*b*_2_. The empirical decay-2 model has four-fitting parameters that increase the chances of a better fit of the water vapor adsorption data on the carbon monoliths compared to the single-parameter pseudo-first-order model.^13^

Comparing the adsorption rate profiles of CO_2_ and H_2_O in Figures 9 and 10, it turns apparent that the adsorption of CO_2_ proceeds faster even though H_2_O is the strongest adsorbate. Thus, it is of utmost interest to elucidate the kinetic performance of both adsorbates when mixed in a gas stream.

**Figure 11:**
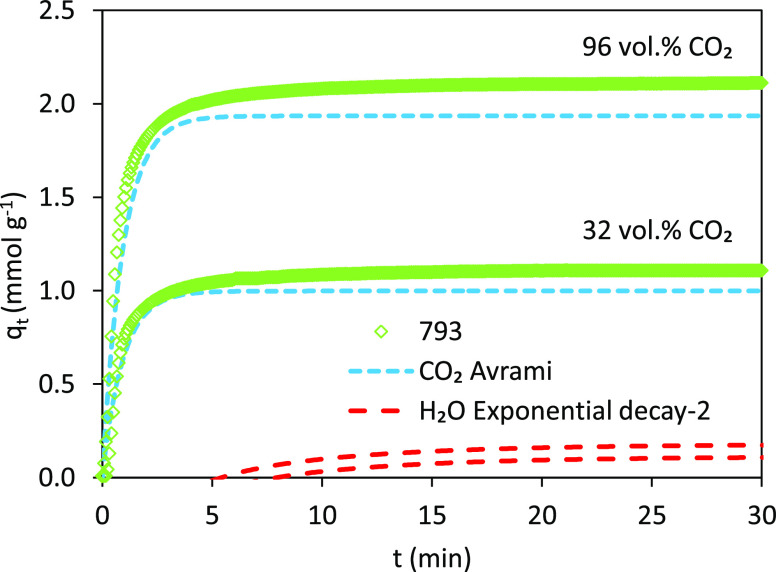
CO_2_ and H_2_O contributions to the adsorption kinetics in 4 vol % H_2_O and 32 or 96 vol % CO_2_ at 50 °C for the carbon monolith 793 (open symbols, experimental data for the total uptake (CO_2_ + H_2_O); dashed blue lines, Avrami’s model predictions for CO_2_; and dashed red lines, exponential decay-2 model predictions for H_2_O).

The dynamic behavior during multicomponent adsorption was split into the CO_2_ and H_2_O contributions, to gain an insight into the performance of adsorbents in the presence of humid CO_2_ streams. To do so, the Avrami and exponential decay-2 model parameters obtained for the individual adsorption of CO_2_ and H_2_O and the corresponding uptakes at each partial pressure were used. It has to be borne in mind that Avrami’s kinetic constant *k*_A_, expressed in min^–1^, is not related to the initial concentration of the adsorbate and H_2_O exponential decay-2 model parameters depend on the relative humidity (RH).^13,60,65^Figure 11 shows the *q*_*t*_ vs *t* plots for the monolith 793 during multicomponent adsorption at two concentrations of CO_2_ in the feed (32 and 96 vol %) and the corresponding predictions of the monocomponent Avrami and exponential decay-2 models. Even though the water concentration in the feed gas kept constant (∼4 vol %) during both multicomponent adsorption experiments, the presence of CO_2_ hindered the uptake of H_2_O_(v)_.

**Figure 12:**
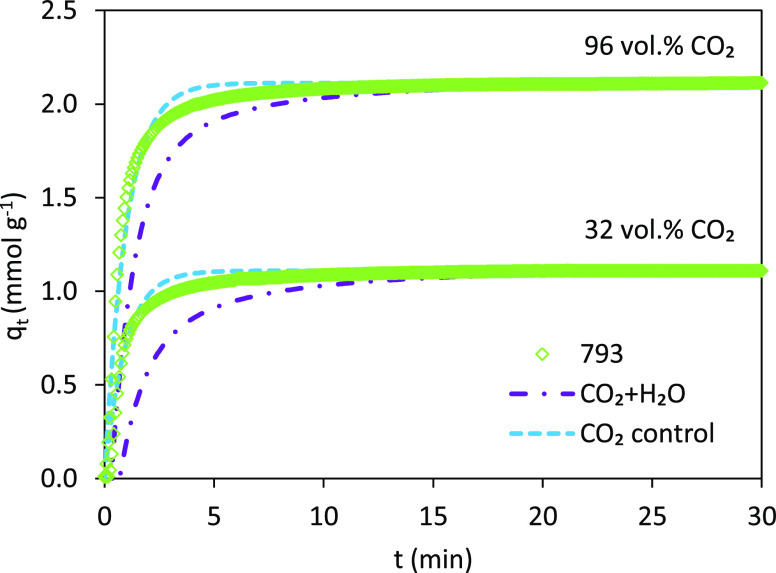
CO_2_ + H_2_O adsorption kinetics analysis for the carbon monolith 793 in 4 vol % H_2_O and 32 or 96 vol % CO_2_ at 50 °C (open symbols, experimental data; dashed blue lines, CO_2_ kinetics control; and dashed purple lines, CO_2_ and H_2_O summed contributions).

As shown in Figure 11, the evolution of the CO_2_ + H_2_O uptakes in the presence of 32 and 96 vol % CO_2_ exhibits two-stage adsorption associated with mass transfer resistances and proper surface adsorption. Adsorption proceeds rapidly at the beginning of the experiment and then slows down with the decreasing of the driving force.^64^ As expected, the uptake is smaller at the lower CO_2_ partial pressure in the feed. The prevalence of CO_2_ adsorption with regard to H_2_O_(v)_ co-adsorption is highlighted in the individual contributions estimated with the models, wherein H_2_O adsorption takes longer times (up to 7.67 min in the experiment with a 32 vol % CO_2_) to initiate compared to CO_2_ adsorption, which occurs instantaneously. The analysis of the CO_2_ + H_2_O uptake has then considered two scenarios: (1) both CO_2_ and H_2_O contribute to the overall kinetics and (2) the CO_2_ uptake controls the overall kinetics. The former implies the addition of the CO_2_ and H_2_O estimated uptakes (see Figure 11), while the latter assumes that the overall kinetics relies on the CO_2_-Avrami model parameters. The values of the experimental uptake considered in the calculations and the associated sum of squared errors (SSE (%)) are listed in Table 15. Figure 12 shows the *q*_*t*_ vs *t* plots for the monolith 793 at two concentrations of CO_2_ in the feed (32 and 96 vol %) for the two scenarios under analysis.

**Table 15:** Values of the Kinetic Model Parameters for the Adsorption Experiments in 4 vol % H_2_O and at Two CO_2_ Partial Pressures at 50 °C

		CO_2_ and H_2_O addition			CO_2_ control	
CO_2_ (vol %)	Sample	*q*_CO_2__ (mmol g^–1^)	*q*_H_2_O_ (mmol g^–1^)	SSE (%)	*q_t_* (mmol g^–1^)	SSE (%)
32	793	1.0	0.1	0.14	1.1	0.03
	932	0.9	0.4	0.21	1.3	0.04
	AM03	0.9	0.5	0.16	1.4	0.05
96	793	1.9	0.2	0.15	2.1	0.04
	932	1.8	0.6	0.20	2.4	0.07
	AM03	2.0	0.7	0.13	2.7	0.07

It can be observed that the first scenario (CO_2_ and H_2_O summed contributions) shows limitations to fit the CO_2_ + H_2_O uptake data during the first half of the experiment in which the adsorption of CO_2_ prevails over H_2_O. On the other hand, when considering the CO_2_ control of the kinetics, the goodness of the fitting significantly enhances as evidenced in Table 15 with the lowest values of SSE (maximum of 0.05 and 0.07% for the experiments feeding 32 and 96 vol % CO_2_, respectively).

As we can infer from Table 15, the goodness of the fittings is consistent for both scenarios with the lowest error values attained for the experiments at the lower CO_2_ concentration. The CO_2_ control scenario better describes the overall kinetics and sample 793 shows the best fittings.

Hence, it can be concluded that the overall adsorption kinetics of humid CO_2_ streams on the carbon monoliths are mainly controlled by the adsorption of CO_2_.

#### Adsorption Mechanism

Mass transfer phenomena impair adsorbate adsorption rate onto porous materials. Kinetic models do not often distinguish the adsorption mechanism, so it is important to explore models that account for the diffusion mechanism involved during the adsorption process and determine the rate-limiting step. Usually, film diffusion, intraparticle diffusion, or both, are the controlling steps of the adsorption rate.^28,63^ Intraparticle diffusion is related to pore volume diffusion (i.e., diffusion takes place in the pores filled with fluid) and surface diffusion (migration through the pore surface, i.e., an adsorbate moves from one available adsorption site to another in various reactions of adsorption and desorption), or both can occur simultaneously.^66,67^

For this purpose, the intraparticle diffusion model sounded on the theory proposed by Weber and Morris was selected to fit the experimental data. This single-resistance model details the process that takes place in the internal pores of the solid. It assumes that intraparticle diffusion is the sole rate-limiting step. The expression in Table 16 derives from Fick’s second law considering the intraparticle diffusivity constant and a small uptake of adsorbate by the adsorbent compared to the concentration in the bulk of the fluid.^68,69^

**Table 16:** Intraparticle Diffusion Model Proposed by Weber and Morris

Kinetic model	Equation	
Intraparticle diffusion	*q*_*t*_ = *k*_i*,d*_*t*^1/2^ + *C*	(68)

Table 16 shows the equation associated with this model, where *t* (min) is the time elapsed from the starting of the adsorption process, *q_t_* (mmol g^–1^) is the amount adsorbed at a given time, *k*_i,*d*_ (mmol g^–1^ min^–1/2^) is the intraparticle diffusion rate constant, and *C* (mmol g^–1^) refers to the boundary layer thickness.

Despite the appearing easiness, the model implementation is not always straightforward to interpret. It is a consequence of the multilinearity of the plots (*q*_*t*_ vs *t*^1/2^), which indicates that more than one stage takes place in the adsorption process. The first linear region corresponds to boundary layer diffusion or external diffusion, the second region is related to the intraparticle diffusion, and the third region is ascribed to the final equilibrium stage. The uncertainty in determining the linear segments translates to the calculation of the slopes and intercepts and, consequently, leads to a loss of accuracy in the estimation of the diffusion coefficients. Besides, valuable information would be lost if the elapsed time from film to intraparticle diffusion, and the transitions that occur between successive intraparticle diffusion regimes were not properly identified.

For this reason, we have used a statistical method called piecewise linear regression (PLR), previously reported by Malash et al.,^52^ to establish the start and end of each linear segment and to identify the number of linear segments. It avoids subjective decisions and the associated errors.

**Figure 13:**
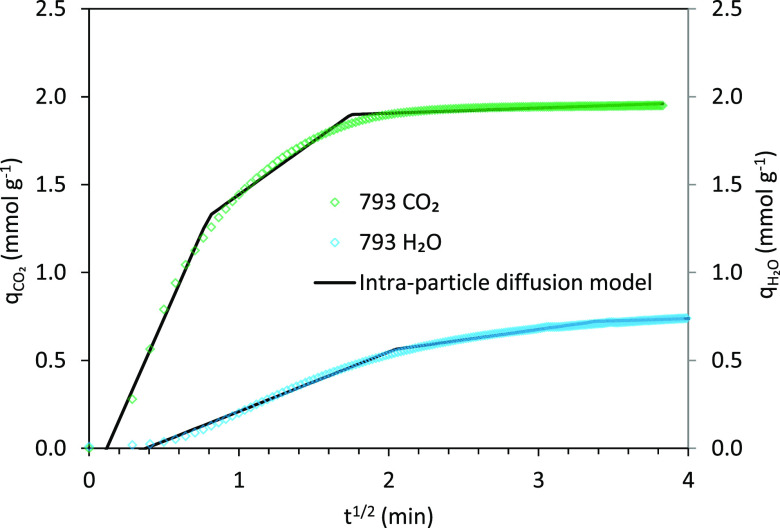
Fittings of the intraparticle diffusion model to the single adsorption of CO_2_ and H_2_O on carbon monolith 793.

If intraparticle diffusion is the only rate-limiting step, the plot should afford a straight line that passes through the origin; meanwhile, in our study, we have identified three main regions in the CO_2_ and H_2_O uptake plots (see Figure 13). These curve-shaped plots account for the following mechanism: the adsorption process starts with the diffusion of the adsorbate (CO_2_ or H_2_O) across the bulk gas/vapor phase to the outer surface of the carbon monoliths along with, in the case of H_2_O, the formation of water vapor clusters around oxygen surface functionalities present on the carbon surfaces. This first segment correlates with the boundary layer diffusion of the adsorbate (CO_2_ or H_2_O). The second corresponds to the gradual CO_2_ or H_2_O adsorption due to intraparticle diffusion toward inner sites (i.e., micropores), where intraparticle diffusion is the rate-limiting step. The last one is assigned to the final equilibrium stage wherein intraparticle diffusion starts to slow down due to saturation of active sites.^70,71^

As expected, the CO_2_ and H_2_O adsorption kinetics on the carbon monoliths depicted completely different plots for both adsorbates and the steepest curves correspond to CO_2_.^6^ In Figure 13, it is observed that the linear fittings of the second and third stages do not pass through the origin and this divergence might be because of the difference in the mass transfer rate between the initial and final adsorption stages as indicated by other authors.^54,72^ Therefore, intraparticle diffusion is not the single rate-limiting mechanism in the adsorption process and film diffusion likewise contributes to the CO_2_ and H_2_O adsorption kinetics on the honeycomb carbon monoliths.^28,73^

The values of the intraparticle diffusion model parameters, the corresponding correlation coefficients (*R*^2^), and the associated sum of squared errors (SSE (%)) are listed in Table 17. These parameters were determined from the fittings of the model to the linear intervals of the uptake profiles identified with the PLR method.

**Table 17:** Intraparticle Diffusion Model Parameters from the Fittings of the Single CO_2_ and H_2_O_(v)_ Adsorption on the Carbon Monoliths^a^

	Sample	Intraparticle diffusion model parameters						
		*k*_i,1_	*k*_i,2_	*C*_2_	*k*_i,3_	*C*_3_	SSE (%)	*R*^2^
CO_2_	793	1.94	0.61	0.83	0.03	1.85	0.02	0.992
	932	1.29	0.43	1.03	0.03	1.80	0.01	0.998
	AM03	2.03	0.61	0.87	0.04	1.91	0.02	0.997
H_2_O	793	0.34	0.12	0.32	0.03	0.64	0.01	0.998
	932	0.41	0.16	0.37	0.04	0.78	0.01	0.997
	AM03	0.39	0.15	0.40	0.03	0.78	0.01	0.997

a *k*_i,*d*_ in mmol g^–1^ min^–1/2^; *C* in mmol g^–1^.

Analyzing the parameters of the first and second linear regions, the intraparticle diffusion rate constant values of the second linear region *k*_i.2_ are inferior for both adsorbates and all of the carbon monoliths.^63,70^ Thus, the diffusion of the adsorbate from the outer surface of the carbon monoliths into the micropores governs the rate of the adsorption.

Extrapolation of the linear fittings back to the y-axis gives the intercepts (*C*), which account for the boundary layer thickness, i.e., the larger the intercept, the greater the boundary layer effect on retarding intraparticle diffusion.^74,75^

Attending to the fittings of CO_2_, the samples with the larger surface areas, 793 and AM03, exhibited higher overall external mass transfer *k*_i,1_ and pore diffusion *k*_i,2_ rates. Slower diffusion may be ascribed to oxygen functional groups concentrated at the entrance of the pores that hinder the diffusion of CO_2_ into the pores.^67^ As expected, the lower external mass transfer rate is a consequence of a wider boundary layer (i.e., sample 932).

As can be seen in Table 17, the diffusion rates for H_2_O_(v)_ are lower due to the reduced water vapor partial pressure in the feed compared to the experiments in pure CO_2_ streams. For H_2_O diffusion, the external mass transfer is also influenced by cluster formation related to the oxygen functional groups content on the surface of the carbon monoliths; this phenomenon allows us to stabilize H_2_O_(v)_ inside the pores and attain faster intraparticle diffusion.^20,76^

An adequate description of intraparticle diffusion of multicomponent mixtures results critical in the simulation and design of PSA processes.^43,77^ Nevertheless, the individual contribution of each species in a mixture may vary substantially from the single-component behavior.^78^ For this reason, CO_2_ + H_2_O intraparticle diffusion parameters have been estimated considering that the overall diffusion is a sum of the CO_2_ and H_2_O contributions. Thus, intraparticle diffusion parameters were calculated for each adsorbate separately. The values of the CO_2_ and H_2_O intraparticle diffusion parameters and the associated sum of squared errors (SSE (%)) are listed in Table 18 and Table 19, respectively.

**Table 18:** CO_2_ Intraparticle Diffusion Model Parameters for the Adsorption Experiments in 4 vol % H_2_O and at Two CO_2_ Partial Pressures on the Carbon Monoliths^a^

		Intraparticle diffusion model parameters						
CO_2_ (vol %)	Sample	*k*_i,1_	*k*_i,2_	*C*_2_	*k*_i,3_	*C*_3_	SSE (%)	*R*^2^
32	793	1.08	0.17	0.59	0.01	0.94	0.012	0.995
	932	0.97	0.16	0.52	0.01	0.85	0.008	1.000
	AM03	0.80	0.11	0.56	0.01	0.81	0.014	0.999
96	793	2.05	0.36	1.14	0.02	1.84	0.016	0.996
	932	1.87	0.31	1.10	0.03	1.70	0.014	0.999
	AM03	1.60	0.28	1.27	0.03	1.84	0.017	0.999

a *k*_i,*d*_ in mmol g^–1^ min^–1/2^; *C* in mmol g^–1^.

**Table 19:** H_2_O Intraparticle Diffusion Model Parameters for the Adsorption Experiments in 4 vol % H_2_O and at Two CO_2_ Partial Pressures on the Carbon Monoliths^a^

		Intraparticle diffusion model parameters						
CO_2_ (vol %)	Sample	*k*_i,1_	*k*_i,2_	*C*_2_	*k*_i,3_	*C*_3_	SSE (%)	*R*^2^
32	793	0.12	0.02	0.06	1.35 × 10^–3^	0.10	0.001	1.000
	932	0.41	0.16	0.52	5.91 × 10^–3^	0.01	0.004	1.000
	AM03	0.44	0.06	0.31	7.66 × 10^–3^	0.45	0.008	1.000
96	793	0.19	0.05	0.08	2.41 × 10^–3^	0.17	0.002	1.000
	932	0.64	0.11	0.35	8.99 × 10^–3^	0.56	0.005	1.000
	AM03	0.58	0.10	0.47	9.23 × 10^–3^	0.67	0.006	1.000

a *k*_i,*d*_ in mmol g^–1^ min^–1/2^; *C* in mmol g^–1^.

Among the linear intervals attributed to the first and second regions, where adsorption is kinetically driven, it can be observed that at any CO_2_ partial pressure, the intraparticle diffusion rate constant *k*_i,2_ is again inferior.^63,70^ This means that joint CO_2_ and H_2_O diffusion through the microporosity of the carbon monoliths is the rate-controlling step.

The CO_2_*k*_i,1_ and *k*_i,2_ values increased with the CO_2_ partial pressure in the feed that enhances the driving force of the CO_2_ to move from the bulk onto the surface and then into the porosity of the carbon monolith.^79,80^ With regard to the water vapor effect on the CO_2_ diffusion, the lower values of CO_2_*k*_i,2_ compared to those of pure CO_2_ (see Table 17) indicate that the presence of water vapor hinders to some extent the transport of CO_2_ through the narrow microporosity.^78^ It is important to highlight that at the two CO_2_ concentrations, the trends are similar for the three carbon monoliths: carbon monolith 793 shows the highest intraparticle diffusion rate, while AM03 seems the most affected by the presence of humidity.

This scenario also leads to significantly lower H_2_O *k*_i,2_ values compared to the pure component (see Table 17) for a similar concentration of water vapor in the feed (4 vol %), demonstrating the predominant role of CO_2_ diffusion in the intraparticle diffusion stage at the two CO_2_ partial pressures considered. This is particularly remarkable for the carbon monolith 793 and highlights its suitability for capturing CO_2_ at the evaluated conditions, representative of cement flue gas.

## Conclusions

The potential of three honeycomb carbon monoliths for their application to humid postcombustion CO_2_ capture from cement-industry flue gases has been explored. Adsorption experiments were conducted to assess the adsorption equilibrium and the kinetics of CO_2_ and H_2_O on the selected carbon adsorbents, under different scenarios representative of postcombustion capture (50 °C and two CO_2_ partial pressures).

The narrow microporosity present on the carbon monoliths allows us to selectively adsorb CO_2_ at partial pressures representative of cement flue gas. From the three carbon monoliths, AM03 showed a more developed micropore network that translated into the highest adsorption capacity of pure CO_2_ and H_2_O.

From the point of view of the kinetics study, the three carbon monoliths present fast adsorption of CO_2_ from a CO_2_/H_2_O stream. The dynamic adsorption of single CO_2_ and H_2_O can be adequately described by the Avrami’s and the exponential decay-2 models, respectively. When a flue gas with 4 vol % H_2_O_(v)_ is considered, overall adsorption kinetics are, however, governed by CO_2_. The fittings of the experimental data to the intraparticle diffusion model revealed that gradual CO_2_ and H_2_O diffusion toward the inner sites (i.e., micropores) was the rate-limiting step. Regarding the effect of water vapor on CO_2_ diffusion, it is important to highlight that at the two CO_2_ concentrations evaluated, the trends were similar for the three carbon monoliths. Carbon monolith 793 showed the highest intraparticle diffusion rate while AM03 seemed the most affected by the presence of humidity.

Under the flue gas conditions evaluated, there exists competitive CO_2_ and H_2_O adsorption; however, in the case of carbon monolith 793, due to its intrinsic characteristics, the adsorption of CO_2_ is little affected (thermodynamically and kinetically) by H_2_O. Therefore, it is a suitable carbon monolith for capturing CO_2_ from cement-industry flue gas streams under the evaluated conditions (∼1 mmol CO_2_ g^–1^ of adsorption capacity and favorable kinetics in 32 vol % CO_2_ and 4 vol % H_2_O_(v)_, at 50 °C and 101.3 kPa).

## Supporting Information Available

CO_2_ and H_2_O TPD profiles; desorption profiles of CO_2_ (*m*/*z* 44), H_2_O (*m*/*z* 18), and N_2_ (*m*/*z* 28); total mass uptake vs time (desorption step) for multicomponent adsorption; and CO_2_*m*/*z* signal normalized (PDF)
