# Supplementary material for: Experimental Study on the Kinetics of CO2 and H2O Adsorption on Honeycomb Carbon Monoliths under Cement Flue Gas Conditions
Source: ACS Sustain Chem Eng. 2022 Jan 31;10(6):2107–24. doi: 10.1021/acssuschemeng.1c07213 (PMC8851587; doi:10.1021/acssuschemeng.1c07213)
Supplement: Supplementary file 1 — sc1c07213_si_001.pdf [file sc1c07213_si_001.pdf]

Supporting Information

Experimental Study on the Kinetics of CO_2 and H_2O Adsorption on Honeycomb Carbon Monoliths at Cement Flue Gas Conditions

Nausika Querejeta[1], Fernando Rubiera[1] and Covadonga Pevida[1,*]

[1]Instituto de Ciencia y Tecnología del Carbono, INCAR-CSIC, c/ Francisco Pintado Fe, 33011 Oviedo, Spain

Corresponding author:

*E-mail: cpevida@incar.csic.es

Number of pages: 5

Number of figures: 4

Table of contents

Temperature Programmed Desorption (TPD) tests S2

Figure S1. TPD profiles: (a) CO_2 and (b) CO evolution for 793, 932 and AM03. .... S2

CO_2 desorption tests S3

Figure S2. Desorption profiles for sample 793: (a) CO_2 (m/z 44), H_2O (m/z 18), and (b) N_2 (m/z 28) profiles. S3

Figure S3. Total mass uptake vs. time (desorption step) for multicomponent adsorption on 793 honeycomb carbon monolith. S4

Figure S4. CO_2 m/z signal normalized for sample 793. .... S5

Temperature Programmed Desorption (TPD) tests

Temperature programmed desorption (TPD) tests were carried out in a thermogravimetric analyzer, Setaram TGA92, coupled to an OmnistarTM mass spectrometer from Pfeiffer Vacuum. Around 70 mg of carbon sample were placed in an aluminum crucible (170 μL) and heated from ambient temperature to 1000 °C (heating rate of 15 °C min^{-1}) under flowing argon (50 cm^3 min^{-1}). Before these measurements, calibration tests with calcium oxalate were carried out. Upon heating in an inert atmosphere, the oxygen surface complexes of carbonaceous materials decompose, releasing CO_2 and CO. CO_2 results from the decomposition of carboxyls, lactones, and anhydrides, while CO comes from anhydrides, phenols, carbonyls, quinones, and pyrones. In the mass spectrometer, the mass to charge (m/z) values 18, 28, and 44 were selected to monitor the evolution of H_2O, CO, and CO_2, respectively. Scientific data analysis and graphing software helped with the fitting of the TPD curves with GaussianAmp peaks. There was no baseline subtraction.

Figure S1 shows the TPD plots (CO_2 and CO evolution) for the honeycomb carbon monoliths.

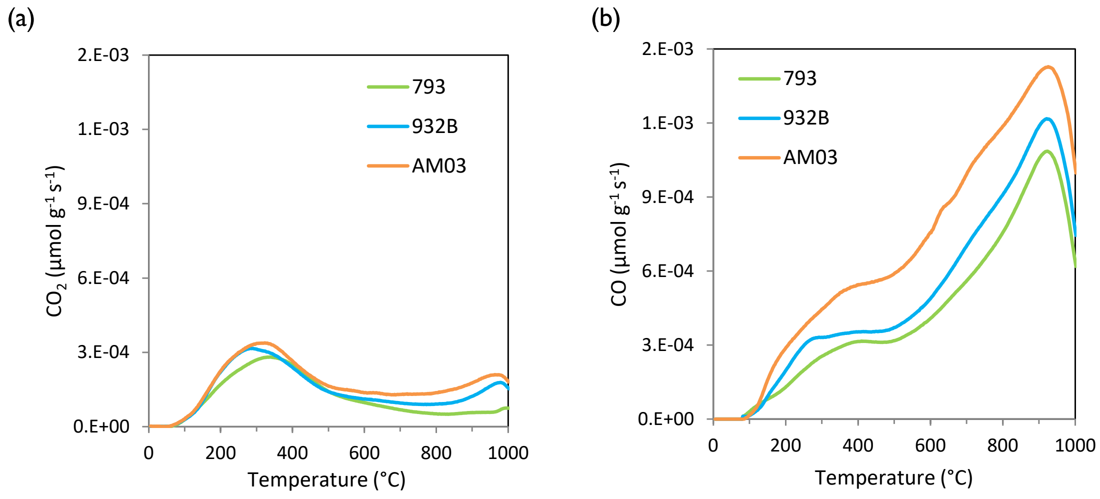

Figure S1. TPD profiles: (a) CO_2 and (b) CO evolution for 793, 932 and AM03.

Overall, the main peak in the CO_2 profiles appears at around 330 °C and it is associated with less acidic carboxylic groups. The large tail in the CO_2 profile that leads to a peak at ~ 568 °C indicates the presence of peroxides [1] and the second main peak located at 1036 °C is assigned to more stable oxygenated groups, such as lactones [2,3].

Likewise, the main peak of CO desorption takes place at 800 °C and is ascribed to the evolution of carbonyls and quinones; at 937 °C, CO desorption continues due to the decomposition of pyrone and chromene groups, whose contributions are difficult to isolate [2]. Besides, the appearance at lower temperatures of other contributions may be due to the thermal decomposition of carbonyl groups in α-substituted ketones and aldehydes [3].

On the other hand, desorption of oxygen groups in the form of CO and CO_2 continues above 1000 °C.

CO_2 desorption tests

The CO_2 adsorption capacity was determined from the amount of CO_2 desorbed to show that during the multicomponent tests on the carbon monoliths the CO_2 uptake reaches the equilibrium even in the presence of a small concentration of water vapor.

By coupling an Omnistar™ mass spectrometer from Pfeiffer Vacuum to the thermogravimetric analyzer, the mass to charge (m/z) 44, 18, and 28 were monitored to account for the evolution of CO_2, H_2O and N_2 during the desorption step following the adsorption step in the multicomponent experiments. The desorption was conducted by heating the sample from 50 °C and 200 °C at a heating rate of 15 °C min^{-1} at a nitrogen flow rate of 100 mL min^{-1}.

Herein, the performances of the honeycomb monolith 793 will be shown in the Figures for illustrative purposes. Figure S2(a) and S2(b) show the m/z 44, 18, and 28 signals evolution during the desorption step corresponding to an experiment feeding a ternary gas mixture (32 vol.% CO_2, 4 vol.% H_2O, N_2 balance) at 50 °C and atmospheric pressure on sample 793. Figure S2(a) shows the CO_2 desorption profile wherein a well-defined peak prevails in the first minutes of the desorption step and then the desorption of CO_2 slows down and there is a large tail until the end of the experiment when the regeneration is completed. The desorption of H_2O is delayed and starts at a later stage. Figure S2(b) shows the N_2 profile that tends to a plateau given that it is the sweeping gas in the desorption stage.

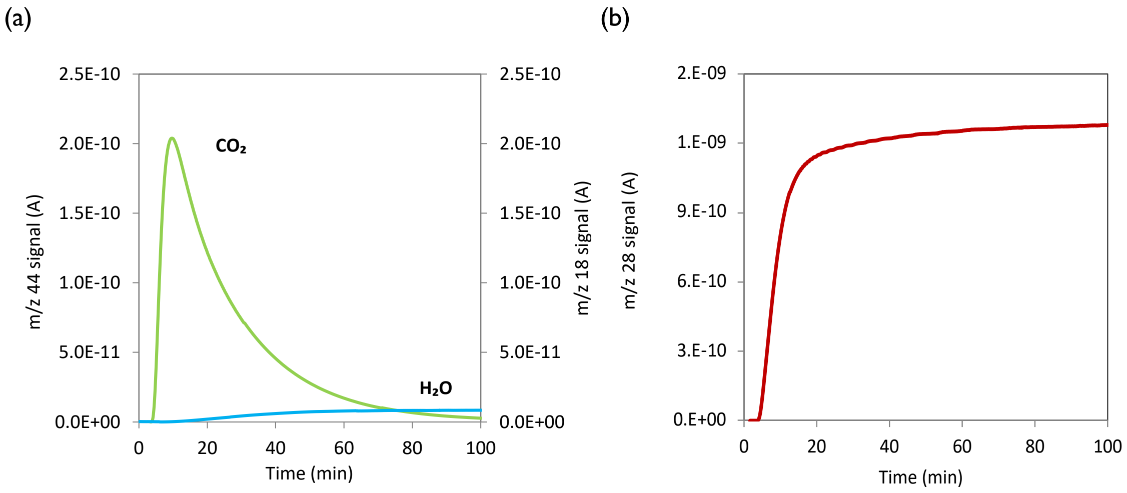

Figure S2. Desorption profiles for sample 793: (a) CO_2 (m/z 44), H_2O (m/z 18), and (b) N_2 (m/z 28) profiles.

On the other hand, the TGA profile during the desorption stage (Figure S3) exhibits a very fast drop in the mass in the first few minutes, due to the rapid desorption of CO_2 (ca. 3.4 wt.% released after ~4 min that corresponds to approximately 62% of the total uptake). Then desorption continues at a slower pace because of the water vapor contribution and reaches a constant mass within ~10 min indicating the full regeneration of the adsorbent (see Figure S3). Although the MS analysis is slightly delayed in time from the TGA, there is a good correspondence between the results in Figures S2 and S3.

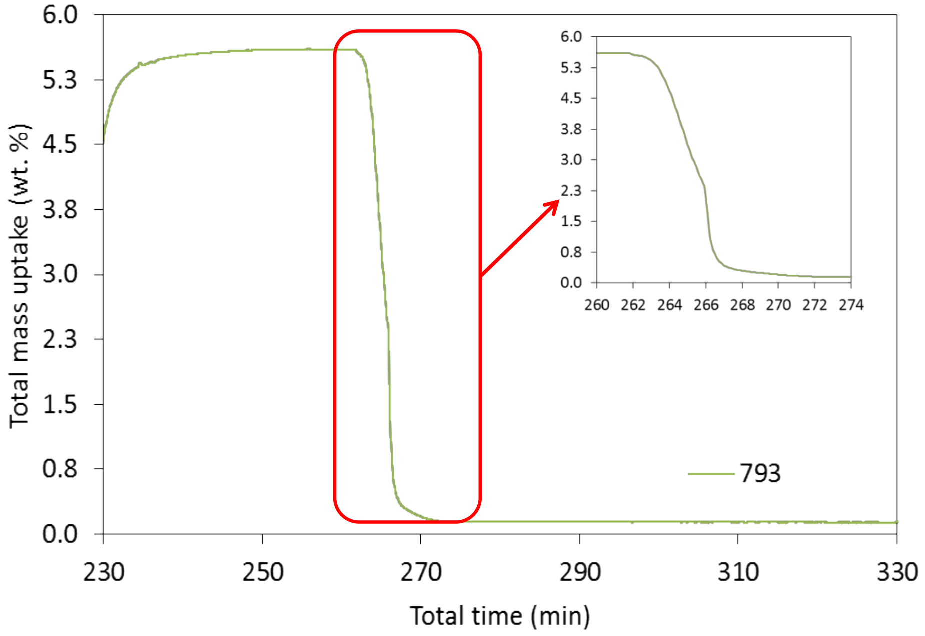

Figure S3. Total mass uptake vs. time (desorption step) for multicomponent adsorption on 793 honeycomb carbon monolith.

The above-mentioned confirms that over the first 4 min of the desorption step only CO_2 is desorbed. Therefore, the amount of CO_2 desorbed over that time frame indicates the extent of the CO_2 adsorption. Calcium oxalate calibration was used to quantify CO_2.

All the intensities of the peaks were expressed in relative terms to maximum and minimum values and normalized to the maximum intensity (N_2 signal) with the following expression:

$$\text{Normalized } [A]CO_2 = \frac{Ion\ current[A]CO_2 - \min[A]CO_2}{\text{Ion current}[A]N_2} \times \max[A]CO_2\ [Ar] \qquad (1)$$

where $Ion\ current[A]CO_2$ and $Ion\ current[A]N_2$ are the intensities of CO_2 (m/z 44) and N_2 (m/z 28) at time t (A), $\min[A]CO_2$ and $\max[A]CO_2\ [Ar]$ are the minimum intensity value of m/z 44 and the maximum intensity value of m/z 44 signal normalized by the linear regression (A).

Making the correspondence between the integrated area (A·s) within the selected time range and the quantities of CO_2 from the calcium oxalate calibration we estimated the amount of CO_2 desorbed. Figure S4 illustrates the normalized m/z 44 signal that corresponds to the CO_2 desorbed from sample 793. The area below the curve for the first 4 min led to a final number of 1.02 mmol g^{-1} of CO_2 desorbed that equals the CO_2 uptake at equilibrium at 50 °C and a CO_2 partial pressure of 32.1 kPa for sample 793.

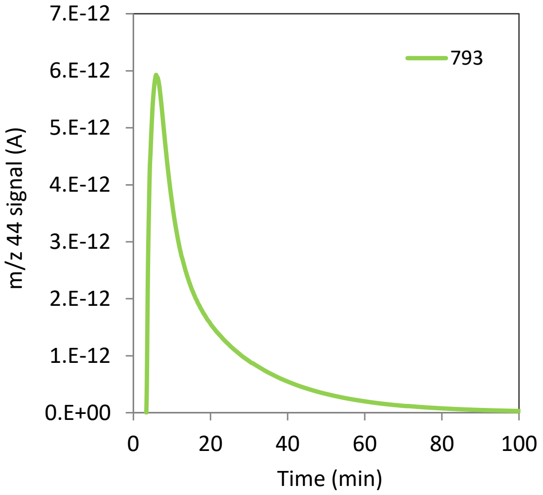

Figure S4. CO_2 m/z signal normalized for sample 793.

These results show that the amount of CO_2 desorbed matches the CO_2 adsorption at equilibrium at the corresponding partial pressure and temperature, and confirm that the CO_2 uptake prevails during the adsorption stage when the relative humidity of the feed is low.

References

1. Zielke, U.; Hüttinger, K. J.; Hoffman, W. P. Surface-oxidized carbon fibers: I. Surface structure and chemistry. *Carbon* **1996**, *34*, 983–998, DOI: 10.1016/0008-6223(96)00032-2.

2. Figueiredo, J. L.; Pereira, M. F. R.; Freitas, M. M. A.; Órfão, J. J. M. Modification of the surface chemistry of activated carbons. *Carbon* **1999**, *37*, 1379–1389, DOI: 10.1016/S0008-6223(98)00333-9.

3. Figueiredo, J. L.; Pereira, M. F. R.; Freitas, M. M. A.; Órfão, J. J. M. Characterization of Active Sites on Carbon Catalysts. *Industrial & Engineering Chemistry Research* **2007**, *46*, 4110–4115, DOI: 10.1021/ie061071v.
